# Microencapsulated probiotic *Lactiplantibacillus plantarum* and/or *Pediococcus acidilactici* strains ameliorate diarrhoea in piglets challenged with enterotoxigenic *Escherichia coli*

**DOI:** 10.1038/s41598-022-11340-3

**Published:** 2022-05-03

**Authors:** Pawiya Pupa, Prasert Apiwatsiri, Wandee Sirichokchatchawan, Nopadon Pirarat, Teerawut Nedumpun, David J. Hampson, Nongnuj Muangsin, Nuvee Prapasarakul

**Affiliations:** 1 Department of Microbiology, Faculty of Veterinary Science, Chulalongkorn University, Bangkok, 10330 Thailand; 2 College of Public Health Sciences, Chulalongkorn University (CPHS), Bangkok, Thailand; 3 Department of Pathology, Faculty of Veterinary Science, Chulalongkorn University, Bangkok, 10330 Thailand; 4 School of Veterinary Medicine, Murdoch University, Perth, 6150 Australia; 5 Department of Chemistry, Faculty of Science, Chulalongkorn University, Bangkok, 10330 Thailand; 6 Diagnosis and Monitoring Animal Pathogens Research Unit, Chulalongkorn University, Bangkok, 10330 Thailand

**Keywords:** Biotechnology, Microbiology, Diseases

## Abstract

*Lactiplantibacillus plantarum* (strains 22F and 25F) and *Pediococcus acidilactici* (strain 72N) have displayed antibacterial activity in vitro, suggesting that they could be used to support intestinal health in pigs. The aim of this study was to determine if microencapsulated probiotics could reduce the severity of infection with enterotoxigenic *Escherichia coli* (ETEC) in weaned pigs. Sixty healthy neonatal piglets were cross-fostered and separated into five groups. Piglets to be given the microencapsulated probiotics received these orally on days 0, 3, 6, 9, and 12. Only piglets in groups 1 and 5 did not receive probiotics: those in groups 2 and 4 received the three microencapsulated probiotic strains (multi-strain probiotic), and piglets in group 3 received microencapsulated *P. acidilactici* strain 72N. After weaning, the pigs in groups 3-5 were challenged with 5 mL (at 10^9^ CFU/mL) of pathogenic ETEC strain L3.2 carrying the *k88*, *staP*, and *stb* virulence genes. The multi-strain probiotic enhanced the average daily gain (ADG) and feed conversion ratio (FCR) of weaned piglets after the ETEC challenge (group 4), whilst supplementing with the single-strain probiotic increased FCR (group 3). Piglets in groups 3 and 4 developed mild to moderate diarrhoea and fever. In the probiotic-fed piglets there was an increase in lactic acid bacteria count and a decrease in *E. coli* count in the faeces. By using real-time PCR, virulence genes were detected at lower levels in the faeces of pigs that had received the probiotic strains. Using the MILLIPLEX MAP assay, probiotic supplementation was shown to reduce pro-inflammatory cytokines (IL-1α, IL-6, IL-8, and TNFα), while group 4 had high levels of anti-inflammatory cytokine (IL-10). Challenged piglets receiving probiotics had milder intestinal lesions with better morphology, including greater villous heights and villous height per crypt depth ratios, than pigs just receiving ETEC. In conclusion, prophylactic administration of microencapsulated probiotic strains may improve outcomes in weaned pigs with colibacillosis.

## Introduction

Pigs are stressed by a variety of factors at weaning, including fundamental changes in gastrointestinal physiology, microbiology, and immunology^1^, and a need for acclimatization to a new environment, adaption to social interaction with new littermates, and challenges associated with withdrawal of sow’s milk and a change in diet^2^. Furthermore, current intensive swine production practises create favourable conditions for the proliferation of infectious agents, particularly bacteria that cause diarrhoeal illness^3^. As a consequence of these circumstances, post-weaning diarrhoea (PWD) remains a major problem in the pig industry.

Infection with enterotoxigenic *Escherichia coli* (ETEC) is the most common cause of PWD, which is characterised by severe anorexia and watery diarrhoea. Piglets can develop hypohydration, lethargy, emaciation, or sudden death after a brief illness^4^. ETEC strains express F4 (K88) adhesive fimbriae, which mediate colonisation of the intestinal mucosa. They can also produce two types of enterotoxins, heat-labile (LT) and heat-stable toxins (STs), which cause hypersecretory diarrhoea^5^.

Antibiotic supplementation in diets may help to alleviate clinical symptoms of PWD, but the routine prophylactic use of in-feed antibiotics to control infections promotes resistance to a wide spectrum of currently available antibiotics^4^. Furthermore, these agents can adversely modify the normal gut microbiota in weaned pigs^6^. Probiotics are live microorganisms that benefit the host’s health^7^, with their main mechanisms of action including preservation of gut integrity, antagonism to pathogenic bacteria, immunological modulation, and general health promotion^8^. As a result, their administration been suggested as an alternative to the use of antibiotics for prevention of ETEC infections.

The lactic acid bacteria (LAB), *Lactiplantibacillus plantarum* strains 22F and 25F and *Pediococcus acidilactici* strain 72N previously have been proposed as useful probiotic bacteria for use in pigs^9–11^. These LAB strains improved pig intestinal health and growth performance throughout the production cycle, giving results similar to those obtained with antibiotic use^12^. Nonetheless, additional in vivo studies are needed to confirm their efficacy in reducing diarrhoea caused by ETEC. Furthermore, many studies in pigs examining the benefits of single-strain and multi-strain probiotics have found discrepancies. Some studies showed that multi-strain were more efficient^13^, while others found that single-strain had more potential^14^. A comparison of single-strain and multi-strain formulations is required in light of these conflicting findings.

It is recommended that to be advantageous to pig health there should be at least 10^6^ colony forming units (CFU) per g or mL of probiotic microbes in the gut^7^. Our previous research has established the effectiveness of double‐coating microencapsulation in preserving LAB properties and survival rates, as well as its potential for probiotic use in livestock farms^12,15^. Therefore, the purpose of the study was to investigate the effectiveness of microencapsulated single-strain and multi-strain LAB against ETEC challenge in pigs post-weaning.

## Results

**Figure 1:**
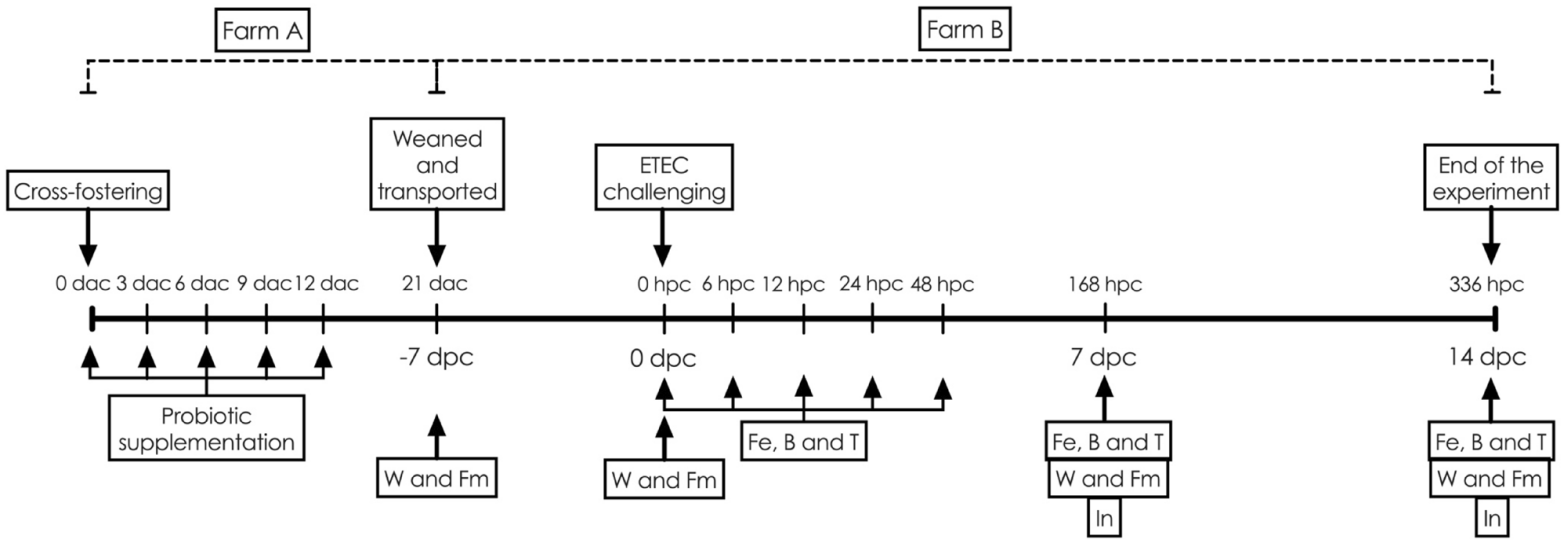
Schematic of experimental designs and sample collection. W = weighing (body weight and feed intake), Fm = Faecal collection for microbial profile analysis, Fe = Faecal collection for ETEC shedding and consistency scoring determination, B = Blood collection, T = Rectal temperature record, and In = Small intestinal collection. dac = day after cross-fostering, hpc = hour post-challenge and dpc = day post-challenge.

Pigs were monitored throughout the experimental period, following the experimental designs (Fig. 1).

### Performance evaluation

**Figure 2:**
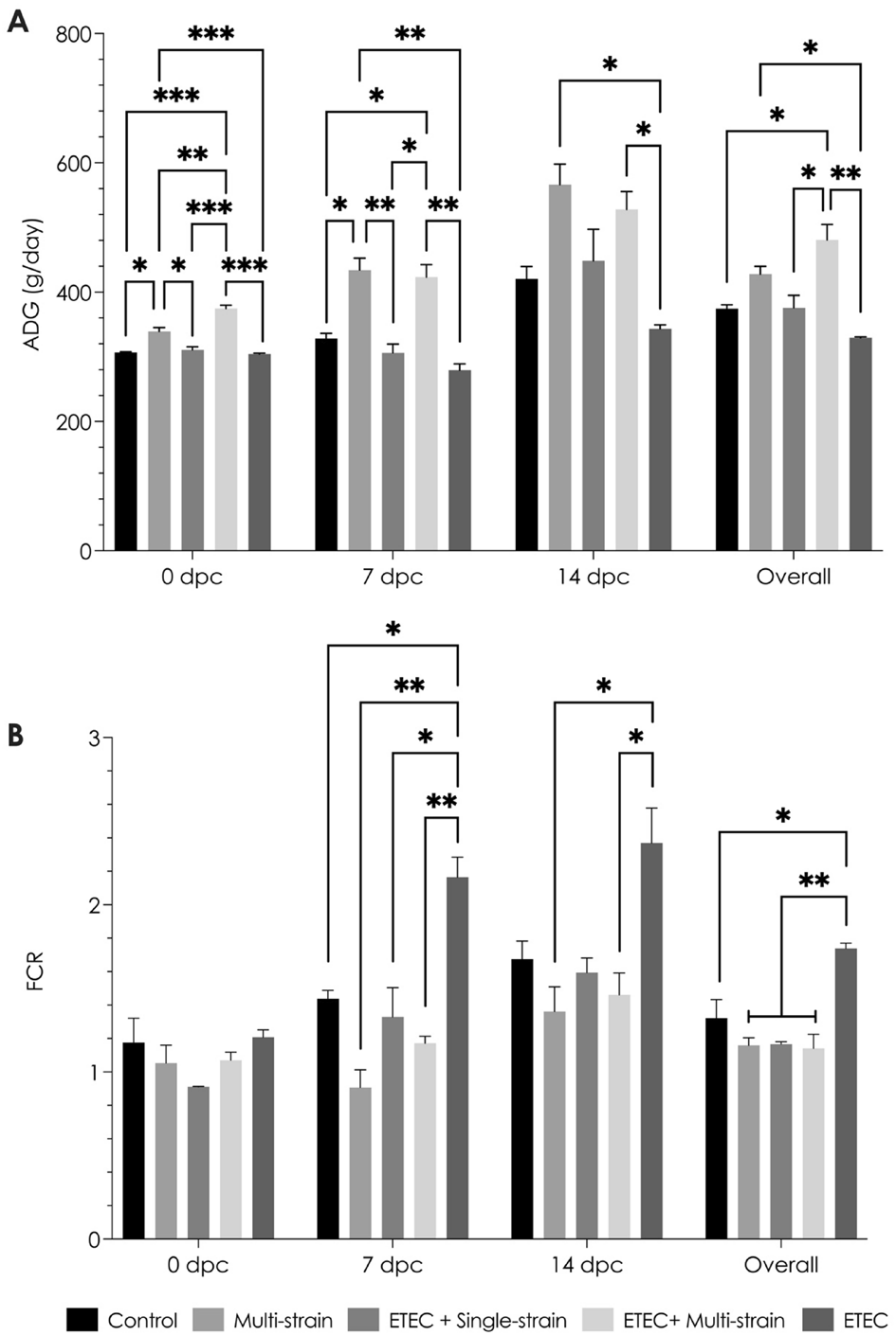
Growth performance of piglets after ETEC challenge (5 × 10^9^ CFU). (**A**)  Average daily gain (ADG); and  (**B**)  Feed conversion ratio (FCR). Control = oral supplementation of sterile peptone water followed by oral administration of sterile peptone water; Multi-strain = oral supplementation of double-coated multi-strain probiotic followed by oral administration of sterile peptone water; ETEC + Single-strain = oral supplementation of double-coated single-strain probiotic followed by oral challenged with ETEC; ETEC + Multi-strain = oral supplementation of double-coated multi-strain probiotic followed by oral challenged with ETEC; and ETEC = oral supplementation of sterile peptone water followed by oral challenge with ETEC. Values are presented as mean ± SEM of all replications in each group. The asterisks represent statistically significant differences (*P < 0.05, **P < 0.01 and ***P < 0.001). dpc = day post-challenge.

The growth performances of piglets are summarized in Fig. 2. When compared to non-probiotic supplemented groups (the control and ETEC groups), probiotic administration significantly increased ADG (*P* < 0.05) before the ETEC challenge (at 0 dpc). Amongst these, the ETEC + single-strain group showed a lower ADG than the multi-strain (*P* < 0.05) and ETEC + multi-strain (*P* < 0.001) groups, which received three probiotic strains. Furthermore, there was no difference in the FCR between the experimental groups. After ETEC challenge, ETEC + multi-strain supplementation improved growth performance (ADG and FCR) at 7 (*P* < 0.01) and 14 (*P* < 0.05) dpc, but ETEC + single-strain supplementation improved FCR only at 7 dpc (*P* < 0.05). In addition, the ETEC + multi-strain fed piglets had a higher ADG at 7 dpc than the ETEC + single-strain group and had similar performance metrics to the multi-strain group (non-ETEC challenge). The ETEC challenge had no effect on ADG (*P* = 0.33) but had a significant effect on FCR (*P* < 0.05) during the entire period (-7 to 14 dpc). In addition, probiotic treatment improved the ADG (*P* < 0.05) and FCR (*P* < 0.01) in ETEC infected piglets. However, as compared to the control group, multi-strain supplementation had no effect on ADG or FCR.

### Diarrhoea score and rectal temperature

#### Faecal consistency score (FCS)

**Figure 3:**
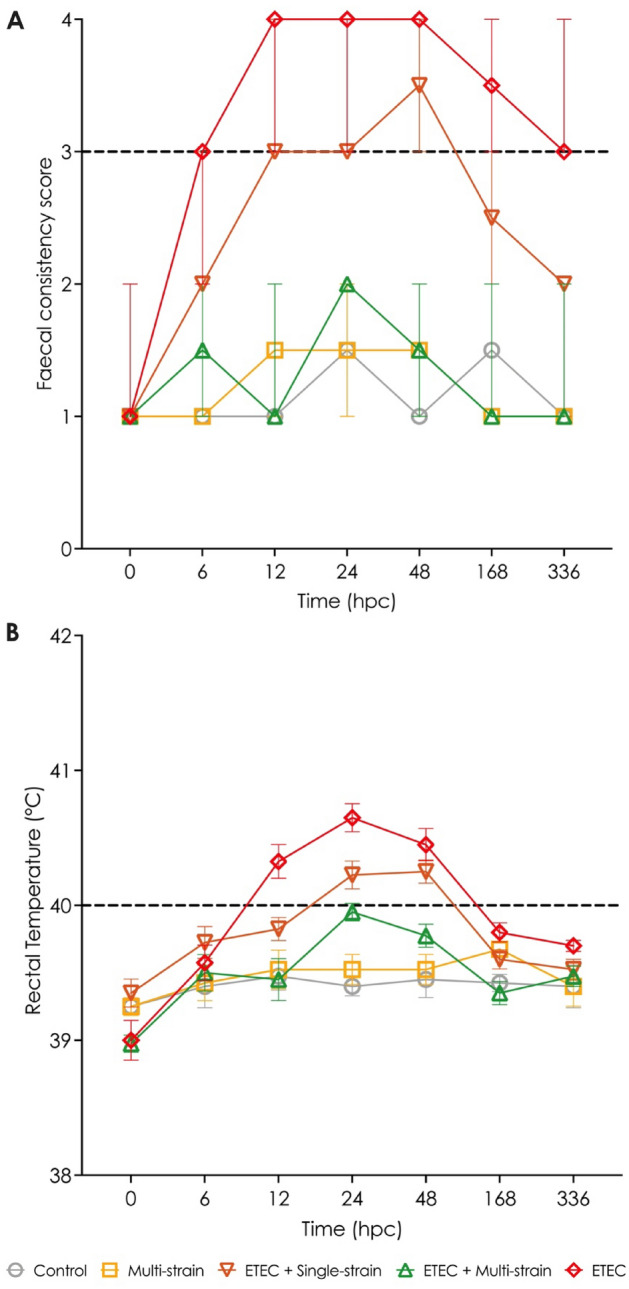
Clinical signs in piglets after ETEC challenge (5 × 10^9^ CFU). (**A**) Faecal consistency score (FCS); and (**B**)  Rectal temperature (RT). Control = oral supplementation of sterile peptone water followed by oral administration of sterile peptone water; Multi-strain = oral supplementation of double-coated multi-strain probiotic followed by oral administration of sterile peptone water; ETEC + Single-strain = oral supplementation of double-coated single-strain probiotic followed by oral challenge with ETEC; ETEC + Multi-strain = oral supplementation of double-coated multi-strain probiotic followed by oral challenge with ETEC; and ETEC = oral supplementation of sterile peptone water followed by oral challenge with ETEC. FCS and RT of all replications in each group are presented as median with range and mean ± SEM, respectively. FCS and RT, which are on or over the dashed line, indicate diarrhoea signs and fever in the piglets. hpc = hour post-challenge.

At 0 hpc (pre-ETEC challenge), no diarrhoea was identified in any piglets in any of the groups, as shown in Fig. 3A. Piglets in the ETEC group showed diarrhoea signs from 6 to 336 hpc after the ETEC challenge, while those in the ETEC + single-strain group had loose stools from 12 to 48 hpc, and recovered before 168 hpc. The FCS of the piglets in the ETEC group was higher (worse status) than in the ETEC + single-strain group. On the other hand, piglets in the ETEC + multi-strain group showed no clinical signs of diarrhoea (FCS less than 3) throughout the experiment, similar to the situation with the groups not challenged with ETEC (the control and multi-strain groups).

#### Rectal temperature (RT)

Before ETEC-challenge, the RT of all piglets was within the usual range of 39 to 39.5 °C, and the piglets in the control and multi-strain groups that were not challenged with ETEC never developed pyrexia. From 12 to 48 hpc, the piglets in the ETEC group showed an increased RT of over 40 °C, and the ETEC challenged pigs receiving the single-strain probiotic also developed pyrexia from 24 to 48 hpc. In both groups, the RT of the piglets returned to normal before 168 hpc. The ETEC + multi-strain supplemented group showed no fever during the testing period following ETEC challenge (Fig. 3B).

### ETEC shedding

**Figure 4:**
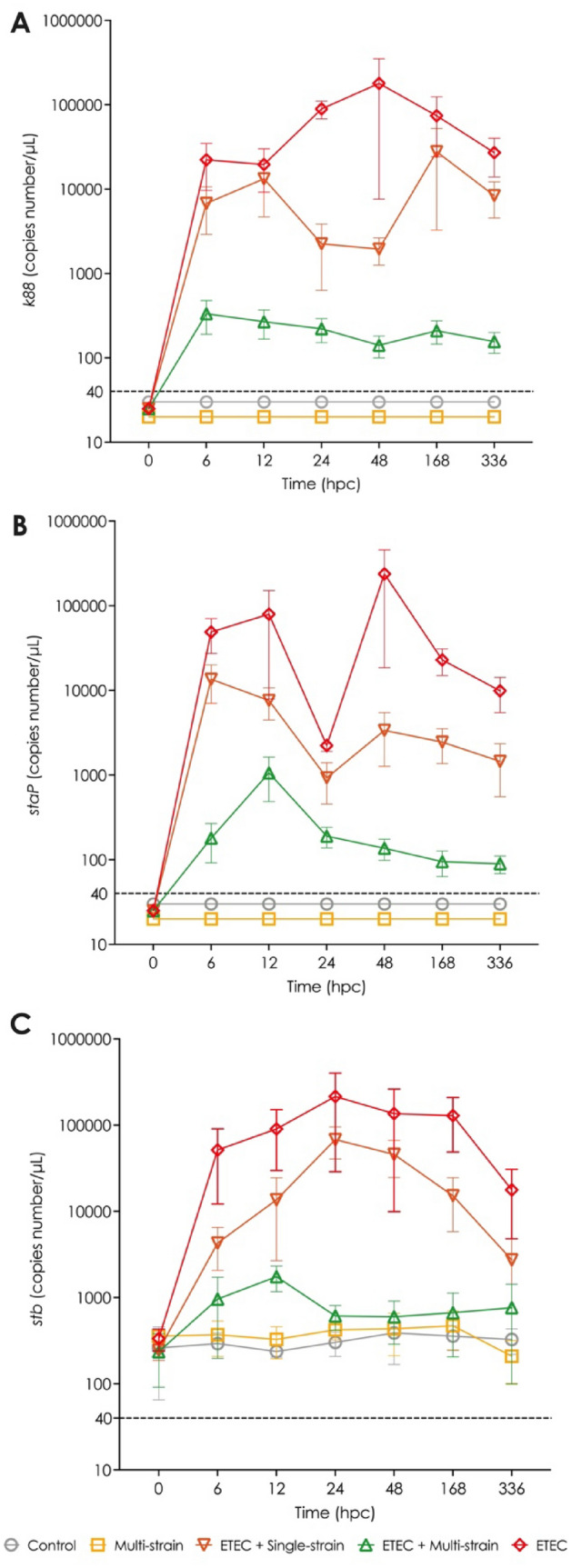
Virulence gene detection in piglets’ faeces after ETEC challenge (5 × 10^9^ CFU). (**A**)  *k88*; (**B**)  *staP*; and (**C**)  *stb*. Control = oral supplementation of sterile peptone water followed by oral administration of sterile peptone water; Multi-strain = oral supplementation of double-coated multi-strain probiotic followed by oral administration of sterile peptone water; ETEC + Single-strain = oral supplementation of double-coated single-strain probiotic followed by oral challenged with ETEC; ETEC + Multi-strain = oral supplementation of double-coated multi-strain probiotic followed by oral challenged with ETEC; and ETEC = oral supplementation of sterile peptone water followed by oral challenge with ETEC. Values are presented as mean ± SEM of all replications in each group. ^abc^Mean values within a time point with different superscript letters are significantly different (*P* < 0.05). Values under the dashed line indicate the under-determination of gene copies number (≤ 40 copies number/μL). hpc = hour post-challenge.

The virulence genes *k88* and *staP* were not detected in the faeces of any piglets prior to the ETEC challenge (at 0 hpc). Furthermore, as shown in Fig. 4A and B, neither gene was detected in the faeces of piglets in the control and multi-strain groups (non-ETEC challenged groups). On the other hand, the *stb* gene was detected at a low level (about < 400 copies number per μL) in faeces samples of the ETEC challenged piglets at 0 hpc and the non-ETEC challenged piglets starting at 0 hpc and continuing throughout the trial period (Fig. 4C). From 6 to 336 hpc, the ETEC challenged groups, especially groups ETEC and ETEC + single-strain, showed higher levels of the three virulence genes in their faeces, although they gradually reduced over time from 168 hpc. Of these two groups, the ETEC + single-strain supplemented piglets tended to have lower levels of the three virulence genes than the piglets in the ETEC group. In contrast to both the former groups, the piglets receiving the ETEC + multi-strain supplement had minimal changes in the number of virulence genes in their faeces following ETEC challenge.

### Faecal microbial counts

Probiotic supplemented piglets (the multi-strain, ETEC + single-strain, and ETEC + multi-strain groups) had significantly higher viable faecal LAB counts (*P* < 0.0001) than the groups not receiving probiotic supplementation (the control and ETEC groups) (Table 1 and Supplementary Fig. S1). The ETEC + single-strain group had a lower LAB count than the other probiotic supplemented groups (*P* < 0.0001). Meanwhile, ETEC challenged pigs had a considerable increase in viable faecal *E. coli,* especially in the ETEC and ETEC + single-strain groups (*P* < 0.0001). On the other hand, the ETEC + multi-strain supplementation significantly reduced *E. coli* numbers in the faeces (P < 0.0001). Piglets in the multi-strain group had a lower faecal *E. coli* count than those in the control group (*P* < 0.0001). The decrease in LAB counts and the increase in *E. coli* counts occurred gradually over the course of the experimental period (*P* < 0.0001). There was also a significant interaction between the experimental group and the time period (*P* < 0.01).

**Table 1:** Faecal LAB and *E. coli* counts (log(CFU/g)) in pigs in each experimental group throughout the experiment.

Experimental group	Period				Mean	Significance^ψ^		
	-7 dpc	0 dpc	7 dpc	14 dpc		E	P	E*P
**LAB**								
Control	9.83 ± 0.04^a^	9.64 ± 0.15^ab^	8.80 ± 0.02^a^	8.62 ± 0.09^ab^	9.22 ± 0.30^A^	< 0.0001	< 0.0001	< 0.01
Multi-strain	10.16 ± 0.05^b^	10.05 ± 0.02^b^	9.41 ± 0.09^b^	9.21 ± 0.06^c^	9.71 ± 0.23^C^			
ETEC + Single-strain	10.08 ± 0.06^ab^	10.06 ± 0.05^ab^	8.92 ± 0.08^ab^	8.73 ± 0.03^ab^	9.45 ± 0.36^B^			
ETEC + Multi-strain	10.14 ± 0.08^ab^	10.07 ± 0.04^b^	9.28 ± 0.12^ab^	8.90 ± 0.03^bc^	9.60 ± 0.30^C^			
ETEC	9.88 ± 0.04^ab^	9.79 ± 0.02^a^	8.73 ± 0.03^a^	8.52 ± 0.04^a^	9.23 ± 0.35^A^			
Mean	10.02 ± 0.07^Z^	9.92 ± 0.09^Z^	9.03 ± 0.14^Y^	8.80 ± 0.12^X^				
***E. coli***								
Control	9.40 ± 0.02^b^	9.59 ± 0.02	9.67 ± 0.02^ab^	9.84 ± 0.04^a^	9.63 ± 0.09^C^	< 0.0001	< 0.0001	< 0.001
Multi-strain	8.77 ± 0.04^a^	9.29 ± 0.06	9.41 ± 0.05^a^	9.55 ± 0.08^ab^	9.26 ± 0.17^A^			
ETEC + Single-strain	9.31 ± 0.03^b^	9.60 ± 0.03	9.96 ± 0.04^ cd^	10.04 ± 0.01^ab^	9.73 ± 0.17^D^			
ETEC + Multi-strain	8.90 ± 0.10^ab^	9.22 ± 0.07	9.75 ± 0.02^bc^	9.83 ± 0.05^ab^	9.43 ± 0.22^B^			
ETEC	9.38 ± 0.07^b^	9.50 ± 0.02	10.02 ± 0.03^d^	10.10 ± 0.02^b^	9.75 ± 0.18^D^			
Mean	9.15 ± 0.13^ W^	9.44 ± 0.08^X^	9.76 ± 0.11^Y^	9.87 ± 0.10^Z^				

^abc^Means with different superscript differ significantly. ^ABCD/WXYZ^Means with different superscript within a column (ABCD) or row (WXYZ) differ significantly.

^ψ^ Significant effects of experimental group (E), period (P) or their interaction (E*P).

### Cytokine evaluation

All groups showed comparable levels of each cytokine concentration before the ETEC challenge (at 0 hpc); however, the pro-inflammatory cytokines IL-1α, IL-6, IL-8, and TNFα increased following the ETEC challenge. In these groups, IL-1α concentration increased significantly from 12 to 336 hpc (*P* < 0.05), as did IL-6 concentration (Fig. 4A and B). In addition, the concentration of IL-8 became elevated from 6 to 48 hpc (*P* < 0.05), before returning to the 0 hpc concentrations by 168 hpc (Fig. 4C). The level of TNFα was markedly higher than in the other groups from 24 to 168 hpc (*P* < 0.05) before declining to starting levels at 336 hpc (Fig. 4D). Except for an increase in IL-8 at 6 hpc and IL-6 at 12 hpc in the ETEC + single-strain supplemented piglets, there was no significant influence of probiotic supplementation (the multi-strain, ETEC + single-strain, and ETEC + multi-strain groups) on serum pro-inflammatory cytokine levels during the experimental period. Furthermore, throughout 12 to 48 hpc, ETEC + multi-strain supplementation significantly increased IL-10 concentrations compared to the multi-strain group (*P* < 0.05), whilst ETEC + single-strain feeding had a similar tendency (*P* = 0.05) (Fig. 4E).

### Pathological analysis

Seven days after ETEC inoculation, the challenged groups (the ETEC + single-strain, ETEC + multi-strain, and ETEC groups) exhibited redness and congestion throughout the small intestine and enlargement of the ileocecal lymph node with the appearance of redness and congestion, which indicated enteritis. Overall, those symptoms were less prominent in the multi-strain group than in the others (Supplementary Fig. S2A). In addition, the severity of the lesions was markedly reduced in pigs killed at 14 dpc, especially in the ETEC + multi-strain group, which had no lesions and looked like non-challenged piglets (the control and multi-strain groups). Similarly, at 14 dpc, there was some improvement in the ETEC + single-strain group’s intestinal lesions. Nevertheless, substantial signs of intestinal disease were still visible in the ETEC group (Supplementary Fig. S2B).

**Figure 5:**
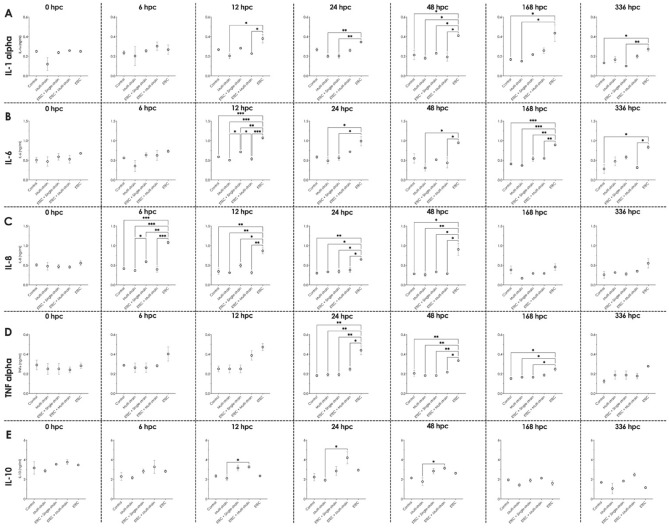
Serum cytokine concentrations at different time point in piglets after ETEC challenge (5 × 10^9^ CFU). (**A**)  IL-1α; (**B**) IL-6; (**C**) IL-8; (**D**) TNFα; and (**E**) IL-10. Control = oral supplementation with sterile peptone water followed by oral administration of sterile peptone water; Multi-strain = oral supplementation of double-coated multi-strain probiotic followed by oral administration of sterile peptone water; ETEC + Single-strain = oral supplementation of double-coated single-strain probiotic followed by oral challenged with ETEC; ETEC + Multi-strain = oral supplementation of double-coated multi-strain probiotic followed by oral challenge with ETEC; and ETEC = oral supplementation of sterile peptone water followed by oral challenge with ETEC. Values are presented as mean ± SEM of all replications in each group. The asterisks represent statistically significant differences (*P < 0.05, **P < 0.01 and ***P < 0.001). hpc = hour post-challenge.

The impact of the ETEC challenge on the jejunal structure was clearly visible in the piglets in the groups ETEC + single-strain and ETEC at 7 and 14 dpc, as shown in Fig. 5A and B, with loss of villi, inflammatory cell infiltration, congestion, and a secretory fluid-filled lumen. However, the ETEC + single-strain supplemented piglets had milder lesions than those in the ETEC group. On the other hand, the ETEC challenge had no effect on the jejunums of the piglets in the ETEC + multi-strain group, which were similar to those of the non-ETEC challenge groups (the control and multi-strain groups). Additionally, piglets in the ETEC + multi-strain group had similar jejunal morphology to those in the multi-strain group at 14 dpc, with exuberant and longer villi observed than the control group (Fig. 5B).

**Figure 6:**
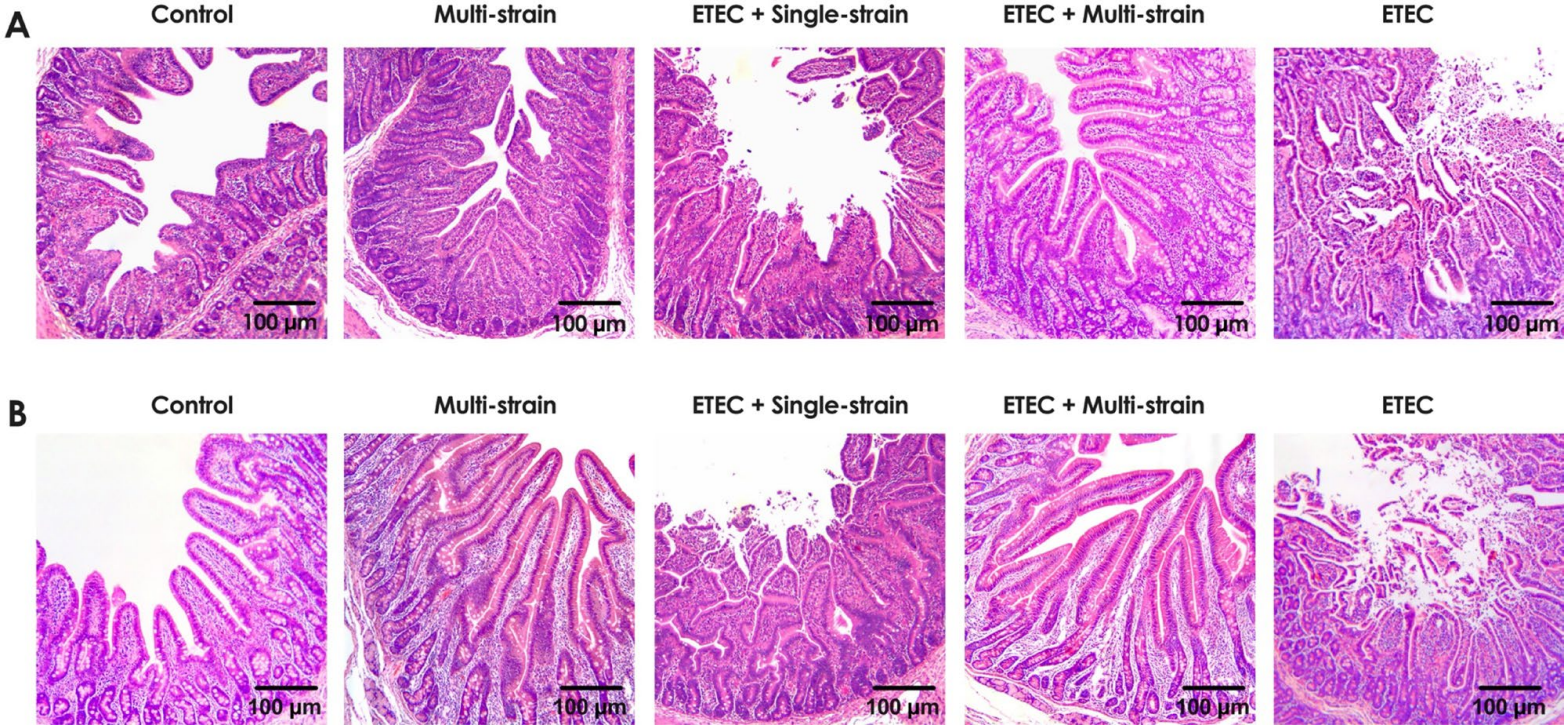
Representative intestinal histo-pathology (jejunum) of piglets after ETEC challenging (5 × 10^9^ CFU). (**A**) 7 days post challenge; and (**B**) 14 days post challenge. Control = oral supplementation of sterile peptone water followed by oral administration of sterile peptone water; Multi-strain = oral supplementation of double-coated multi-strain probiotic followed by oral administration of sterile peptone water; ETEC + Single-strain = oral supplementation of double-coated single-strain probiotic followed by oral challenge with ETEC; ETEC + Multi-strain = oral supplementation of double-coated multi-strain probiotic followed by oral challenge with ETEC; and ETEC = oral supplementation of sterile peptone water followed by oral challenge with ETEC.

Supplementary Fig. S3–S5 shows the histometric effects of the experimental treatment on the small intestine (duodenum, jejunum, and ileum), including VH, CD, and VH:CD ratio at 7 and 14 dpc. The VH and VH:CD ratios were lower after ETEC challenge (*P* < 0.0001). Of the three ETEC challenged groups, the probiotic supplemented piglets in the ETEC + single-strain and ETEC + multi-strain groups had a more favourable VH (*P* < 0.001) and VH:CD ratio (*P* < 0.01). In addition, ETEC + multi-strain supplementation maintained intestinal structure (VH and VH:CD ratio) better than did the ETEC + single-strain supplementation (*P* < 0.0001). Multi-strain supplementation significantly increased the VH and VH:CD ratio in non-ETEC challenged piglets compared to the control group (*P* < 0.01). Interestingly, despite being infected with ETEC, piglets in the ETEC + multi-strain group had better intestinal structure parameters than those in the control group (*P* < 0.05), although the CD did not differ between the groups.

## Discussion

Various experimental challenge models have been developed to study the pathogenesis and means to control porcine PWD resulting from ETEC challenge^16^. In the current study a single oral dose of 5 mL of ETEC strain L3.2 (10^9^ CFU/mL per piglet) given at 7 days after weaning resulted in clinical signs (diarrhoea, fever) and changes in pro-inflammatory cytokine profiles and intestinal morphology consistent with PWD, especially in pigs that had not received probiotics. Effects were greatest at 7 days post-challenge, and had diminished by 14 days. At the same time the numbers of ETEC in the faeces increased. Hence the ETEC strain used and challenge method adopted resulted in a credible model of porcine PWD. According to our findings, the detection ETEC virulence genes (*k88*, *staP*, and *stb*) in faeces was temporally linked to the occurrence of clinical signs. Small numbers of *stb* genes also were detected in the faeces of non-ETEC challenged piglets, but these did not develop clinical signs. This finding is consistent with previous research that has shown that low levels of some virulence genes can be detected in the faeces of healthy pigs^17^. Our study suggests that quantitatively detection of the three virulence genes is a useful means of assessing ETEC shedding and proliferation in weaned pigs.

The high levels of serum pro-inflammatory cytokines (IL-1α, IL-6, IL-8, and TNFα) found in the serum of piglets exposed to ETEC strain L3.2 is likely to have been associated with inflammatory cell recruitment and infiltration^5^; also the cytokines may have induced apoptosis in gut epithelial cells^18^, correlating with the clinical indication of enteritis and a change in intestinal morphology. As a result of these changes, infection with ETEC strain L3.2 reduced pig growth, feed efficiency, and feed consumption in a manner consistent with naturally occurring PWD. The shortening of villi results in a reduction in surface area for nutrient absorption^19^, and immunological activation may also divert resources and energy from growth to immune response and epithelial repair^20^.

In this study, administration of microencapsulated probiotic strains on five occasions between days 0–12 after cross-farrowing resulted in intestinal colonization as assessed by faecal LAB counts at weaning. Subsequently, these probiotic supplementations (single-strain; *P. acidilactici* strain 72N and multi-strain; *Lb. plantarum* [strains 22F and 25F] and *P. acidilactici* strain 72N) were shown to ameliorate the detrimental effects of ETEC challenge (reduced diarrhoea, fever, and faecal ETEC shedding), and particularly so with the multi-strain preparation. The decreased faecal *E. coli* and increased faecal LAB numbers following exposure to the probiotic strains reflected a better gut microbial environment. The study did not attempt to quantify colonization with each of the three inoculated LAB strains, so their relative influences and affects in individual pigs remains uncertain. Nevertheless, overall the beneficial effects of the LAB strains on the faecal microbial profile and on gut health are consistent with findings using other probiotic strains, including *Enterococcus faecium* NCIMB 10415 and multispecies probiotics (*L. acidophilus*, *L. casei*, *Bacillus thermophilum*, and *E. faecium*), which have been shown to reduce the severity of diarrhoea in sucking and weaning piglets challenged with ETEC K88 and *E. coli* F18^+^^19,20^. In addition, supplementation with *L. rhamnosus* GG (ATCC 53103)^21^ and *L. plantarum* CJLP243^22^ has been shown to reduce the pyrexia stage in weaned pigs challenged with ETEC K88ac. In another study, feeding *L. plantarum* CJLP243 to weaned piglets resulted in a reduction in ETEC detection by PCR in faecal samples of challenged pigs. Similarly, a previous study showed that adding *B. licheniformis* and *S. cerevisiae* reduced *E. coli* and increased *Lactobacillus* in the caecal contents of weaned pigs challenged with ETEC K88^23^. On the other hand, another study found that giving *L. rhamnosus* GG (ATCC 53103) did not reduce diarrhoea or faecal *E. coli* F4 levels in piglets^24^.

One way that colonization with probiotic strains may enhance gut health is by exerting antimicrobial activity against pathogenic bacteria, such as the ETEC strain used in the current experiment. For example, they may secrete lactic acid, contributing to an acidic environment in the intestine, or produce bactericidal compounds, inhibiting the growth or adhesion of pathogenic bacteria. In previous in vitro investigations, cell-free supernatants of the live and microencapsulated forms of the three LAB strains used in this study have been shown to inhibit or kill ETEC strains^9,15^. In addition, LAB strains may compete for nutrients or binding sites on the epithelium and so prevent pathogen colonization^8^. Hence our multi-strain probiotic may exert competitive exclusion of pathogenic microbes, whilst favouring beneficial microbes^25^.

Feeding the weaned piglets with our single-strain and multi-strain probiotics modulated the release of serum pro- and anti-inflammatory cytokines after ETEC infection. Similarly, the addition of *L. rhamnosus* GG (ATCC 53103) decreased serum IL-6 in piglets exposed to ETEC K88ac^21^, and the addition of multispecies probiotics (*L. acidophilus*, *L. casei*, *B. thermophilum* and *E. faecium*) decreased TNFα levels in the serum of piglets challenged with *E. coli* F18^+^^20^. Similarly, dietary supplementation with fermented soybean meal (made from soybean meal by fungal and bacterial strain) elevated plasma IL-10 in pigs after ETEC K88 challenge^26^. Nevertheless, some other studies have reported that supplementation of probiotics such as *B. longum* subsp. *infantis* CECT 7210^27^ and *E. faecium* NCIMB 10415^19^ did not impact on serum or plasma cytokine levels (IL-1β, IL-6, and TNFα) of piglets inoculated with ETEC K88. ETEC expressing K88 fimbriae mediate bacterial adhesion to host cells, activating the innate immune and inflammatory responses by engaging the TLR/MyD88/NF-κB signal pathway, contributing to diarrhoea and impaired intestinal barrier integrity. Our probiotic strains may reduce pro-inflammatory cytokine production by reducing ETEC-induced TLR2 expression, which is linked to the down-regulated NF-κB and MAPK signaling pathways in piglets^19,28,29^. The suppression of the cytokine signaling (SOCS) family proteins influences IL-10 production, which is mediated by JAK-dependent activation/phosphorylation of dimeric STAT transcription factors. It is possible that our probiotic strains may have increased levels of transcription factor STAT3, which is involved with IL-10 signaling, causing SOCS3 to decrease expression of the genes encoding pro-inflammatory cytokines^26,30^. Furthermore, after ETEC-challenge, piglets supplemented with the single-strain and multi-strain probiotics had reduced intestinal damage. Similarly, the administration of multispecies probiotics (*L. acidophilus*, *L. casei*, *B. thermophilum*, and *E. faecium*) to piglets challenged with *E. coli* F18^+^^23^ and feeding *B. licheniformis* and *Saccharomyces cerevisiae* to ETEC K88 challenged piglets^20^ had positive effects on VH and VH:CD ratios. On the other hand, administering *L. rhamnosus* GG (ATCC 53103) after *E. coli* F4 inoculation had no effect on intestinal morphology^24^. As previously stated, our probiotic strains may reduce ETEC-induced inflammation by regulating cytokine production, suppressing ETEC growth, and hence decreasing enterotoxin release. Furthermore, the barrier function effect could be another important mode of action of our probiotic strains. These strains may help epithelial cells maintaining their integrity by enhancing the expression of genes involved in tight junction proteins, such as E-cadherin and β-catenin. In addition, they may aid in the restoration of the gut barrier after damage by redistributing the tight junction proteins, zonula occludens (ZO-2), resulting in a tight junction complex reconstruction^8^. Importantly, the use of our single-strain and multi-strain probiotics was beneficial in alleviating the growth retardation associated with ETEC strain L3.2 in weaned piglets, including improving ADG and FCR. As previously reported, supplementation with *L. salivarius*^31^ and *B. longum* subsp. *infantis* CECT 7210^32^ improved the ADG of pigs infected with ETEC F4. On the other hand, administration of *B. longum* subsp. *infantis* CECT 7210^27^ and multispecies probiotics (*L. acidophilus*, *L. casei*, *B. thermophilum*, and *E. faecium*)^20^ did not improve performance parameters after ETEC K88 and *E. coli* F18 + challenge in piglets. In summary, the beneficial effects of our probiotic strains could be due to a variety of mechanisms, including competitive exclusion of pathogenic microorganisms (ETEC), production of antimicrobial substances, improving gut microbiota homeostasis, enhancing immune system regulation, and improving epithelial barrier function^8^. Further studies are required to examine these potential protective mechanisms in more detail, so that their affects can be optimized.

## Conclusions

In conclusion, challenge with ETEC strain L3.2 in weaned pigs caused PWD, which was associated with a pronounced cytokine response, pathological lesions, impaired gut morphology, and changes in LAB to *E. coli* ratio, all of which were associated with poor growth performance. On the other hand, feeding microencapsulated *P. acidilactici* strain 72N (single-strain) and microencapsulated *Lb. plantarum* strains 22F and 25F and *P. acidilactici* strain 72N (multi-strain) before weaning reduced the impact of ETEC challenge, being associated with decreased ETEC detection, modulation of the cytokine response, reduction of intestinal damage and clinical signs, and improved growth performance. The multi-strain probiotic appeared to have a better effect than the single-strain one. Overall, administration of our probiotic strains to neonatal piglets proved to be beneficial in ameliorating PWD resulting from ETEC infection.

## Materials and methods

### Ethics statement

The study was conducted in the Feed Research and Innovation Centre, Charoen Pokphand Foods (CPF) Public Company Limited (PLC.), and in the Thai Food Research Center, Thai Foods Group (TFG) Public Company Limited (PLC.). The experimental protocols and methods in this study were carried out in compliance with the ARRIVE guidelines. The animals utilised in this study were managed in compliance with the guidelines for experimental animals established by the Institute Animal Care and Use Committee of the Feed Research and Innovation Center of CPF (FRIC-ACUP-1707013) and the Thai Food Research Center (6112–01). All LAB and ETEC strains used in this study were approved by Chulalongkorn University's Institutional Biosafety Committee under protocols IBC1831044 and IBC1831045, respectively.

The euthanasia procedures were performed following the guidelines for the euthanasia of animals complied with the American Veterinary Medical Association (AVMA). All pigs were humanely terminated by potassium chloride administration in conjunction with the general anesthetic technique. Briefly, pigs were rendered unconscious by administered pentobarbital sodium by intravenous injection for causing depression of the central nervous system. They were then administered intracardially with potassium chloride, leading to cardiac arrest and death. All efforts were made to minimize the suffering.

### Bacterial preparation and growth conditions

In preliminary testing, the concentrations of bacterial cells, including LAB and ETEC, were assessed using densitometry and the CFU count method.

Selection and preparation of lactic acid bacteria.

Previous in vitro studies demonstrated that *Lb. plantarum* strains 22F and 25F, and *P. acidilactici* strain 72N have probiotic properties such as acid and bile tolerance, lack of antimicrobial-resistance genes using EFSA criteria, have antibacterial properties against *E. coli* and pathogenic *Salmonella*, and demonstrate viral interference against porcine endemic diarrhoea virus^9–11^. Furthermore, among the three LAB strains tested in pigs, *P. acidilactici* strain 72N showed the greatest potential to improve growth performance, microbial profile and gut health^12^. Hence, this strain was chosen as a single-strain probiotic, and all three strains were combined as a multi-strain probiotic.

The probiotic bacteria were kept at -80 °C in De Man, Rogosa and Sharpe (MRS) broth (Becton, Dickinson and Company) containing 20% glycerol. Bacterial strains were cultivated at 37 °C for 18-20 h in MRS medium. *P. acidilactici* strain 72N was harvested by centrifugation (3,000 × g, 4 °C, 10 min), washed, and resuspended in sterile normal saline to achieve a final concentration of 10^9^ CFU/mL for the single-strain experiment^12^. Each of the three LAB strains was harvested by centrifugation, washed twice, and resuspended in normal saline individually, as indicated previously. For the multi-strain probiotic, *Lb. plantarum* strains 22F and 25F, and *P. acidilactici* strain 72N were blended at a ratio of 1:1:1 to obtain a final concentration of 10^9^ CFU/mL.

#### Enterotoxigenic *Escherichia coli* (ETEC)

In a previous study, ETEC strains were isolated from piglets with clinical diarrhoea in commercial swine farms in Petchabun and Lopburi Provinces^33^. Following detection of virulence genes *k88*, *staP* and *stb*, three strains of ETEC, L3.1, L3.2, and VE1 were chosen as potential strains for challenge studies. The pathogenicity of those three strains was assessed in experimentally infected weaner pigs in a pilot study, by comparing faecal consistency score (FCS), rectal temperature (RT), and intestinal pathology (unpublished data). Pigs receiving strain L3.2 had the most severe watery diarrhoea and intestinal lesion. As a result, ETEC strain L3.2 was selected for used as the challenge strain in this study.

ETEC strain L3.2 was kept at -80 °C in Luria–Bertani (LB) broth (Becton, Dickinson and Company) containing 20% glycerol. The strain was cultivated overnight in LB medium at 37 °C. Bacterial cells were pelleted by centrifugation at 3,000 × g for 10 min at 4 °C, then washed and adjusted to 10^9^ CFU/mL in sterile normal saline^21^.

### Microencapsulation of probiotic strains

Probiotic cell suspensions were subjected to double-coating microencapsulation with alginate and chitosan using the spray drying process, as previously reported^15^. In brief, for inner capsulation, a 1.5% (w/v) alginate solution (Sigma-Aldrich) was prepared. Single stain and multi-strain probiotics at 10^9^ CFU/mL were combined separately with the alginate solution at a 1:5 (v/v) ratio. The alginate granules were harvested from the collecting vessel after being atomized through a spray dryer (Mini Spray Dryer B-290, Buchi) with an inlet temperature of 130 °C. The exterior microcapsule was made with a 0.5 percent (w/v) chitosan solution (Union Chemical 1986) in 100 mL of 1 percent (v/v) acetic acid (Merck KGaA). One gram of those powders was mixed with 100 mL of chitosan solution before being atomized in the spray dryer under the same conditions as described. The probiotic-containing double-coated powders were harvested from the collection vessel and stored at room temperature for six months before use in this study.

### Animals and housing

A total of 60 healthy newborn piglets (Large White × Landrace × Durox) were randomly assigned into five experimental groups after cross-fostering (12 pigs per group). By stratified random sampling by gender, all piglets in each group were divided into two replications (6 pigs per replicate pen). Those piglets were obtained and raised until weaning at the CPF Feed Research and Innovation Center's animal experiment site, dubbed Farm A. The piglets with their assigned sows were kept in an environmentally controlled building with an evaporative cooling system. Plastic floor mats, a heated plastic mat cover, a heat light, a feeder for creep feed, and a water nipple were provided in each pen (2 × 2.4 m; 6 piglets per pen). The temperature and humidity in the house were kept at 32 ± 2 °C and 80 percent, respectively. The photoperiod was set to 12 h of light and 12 h of darkness. At weaning at 21 days of age, all piglets were moved to Farm B, an isolation animal building at the Thai Food Research Center. Each experimental group of twelve piglets was housed in an individual room and contained in a pen (2 × 2 m; 6 piglets per pen), which included a heated plastic mat cover, a single heat bulb, a feeder, and a nipple drinker. The housing was ventilated and had a 12-h light cycle, with a temperature of 27 to 28 °C and a humidity of 80%.

### Experimental design, inoculations and sample collection

The piglets were divided into 5 groups: Group 1 (control)—no probiotic supplementation and no ETEC challenge; Group 2 (multi-strain)—supplemented with spray-dried *Lb. plantarum* strains 22F and 25F and *P. acidilactici* strain 72N, and no ETEC challenge; Group 3 (ETEC + single-strain)—supplemented with spray-dried *P. acidilactici* strain 72N and challenged with ETEC strain L3.2; Group 4 (ETEC + multi-strain)—supplemented with spray-dried of *Lb. plantarum* strain 22F, 25F and, *P. acidilactici* strain 72N and challenge with ETEC strain L3.2; Group 5 (ETEC)—no supplementation and challenge with ETEC strain L3.2, respectively. The groups and treatments are summarized in Table 2, and an overview of the experimental protocol is shown as Fig. 1.

**Table 2:** Initial average bodyweight and summary of the experimental groups.

No	Experimental group	Initial average BW (kg)^ψ^	Probiotic supplementation			ETEC challenge
			*P. acidilactici* strain 72 N	*Lb. plantarum* strain 22F	*Lb. plantarum* strain 25F	
1	Control	1.93 ± 0.06	–	–	–	–
2	Multi-strain	1.93 ± 0.06	√	√	√	–
3	ETEC + Single-strain	1.98 ± 0.05	√	-	-	√
4	ETEC + Multi-strain	1.97 ± 0.05	√	√	√	√
5	ETEC	1.92 ± 0.08	–	–	–	√

√ and—represent with and without supplementation or challenge, respectively.

^ψ^ Values are presented as mean ± SEM.

The designated groups of piglets were either given 3 mL sterile peptone water (Becton, Dickinson and Company, Maryland, USA) or 1 g of the appropriate microencapsulated probiotics suspended in 3 mL sterile peptone water orally via sterile syringe on days 0, 3, 6, 9, and 12 days after cross-fostering (dac). The piglets were allowed to suckle sow’s milk conventionally until weaning at 21 dac when they were relocated to Farm B and allowed to acclimatize for a week. They received fresh water ad libitum and were fed a basal weaner diet that did not contain antimicrobials and that met the NRC guidelines (Supplementary Table S1). Their faeces were tested for ETEC by PCR amplification of the *k88* gene at 21 dac^34^.

On day 7 after transfer to farm B, piglets in the control and multi-strain group were orally administered with 5 mL of sterile peptone water, while the others received 5 mL of ETEC strain L3.2 (10^9^ CFU/mL per piglet). Subsequently, throughout the 14 days of the experiment, the clinical signs of all the piglets were monitored twice a day (at 8 AM and 4 PM), and the piglets identified as moribund were euthanised. Faecal samples were collected at 0- (before), 6-, 12-, 24-, 48-, 168- and 336-h post-challenge (hpc) to determine ETEC shedding and faecal consistency scores. At the same sampling times, rectal temperatures (RTs) of all pigs were recorded using a thermometer, and mean temperatures for the groups were compared. Temperatures ≥ 40 °C were used to indicate that the piglets had pyrexia^21,22^. Concurrently, blood was collected from 4 pigs (2 males and 2 females) in each experimental group by cranial vena cava venipuncture into a 6-ml heparin-free vacutainer tube (BD Vacutainer) for cytokine analysis. Six piglets (3 males and 3 females) per group were euthanised at 7- and 14-day post-challenge (dpc), and the small intestines (duodenum, jejunum, and ileum) were removed for pathological investigation. Faeces also were collected at -7, 0, 7, and 14 dpc to investigate microbial profile (LAB and *E. coli* numbers). Bodyweight and feed intake of all pigs were monitored weekly for performance evaluation from -7 dpc and used to compute the average daily gain (ADG) and feed conversion ratio (FCR), and the average scores for the groups were recorded.

### Faecal consistency score

A faecal consistency score (FCS) system was used to determine the diarrhoea score of all pigs: 1 indicates normal (well-formed faeces); 2 indicates soft faeces (formed faeces); 3 indicates mild diarrhoea (sloppy faeces); and 4 indicates severe diarrhoea (pasty and liquid faeces). Median scores were used for each group. When the faeces consistency score was ≥ 3, piglets were regarded as having diarrhoea. The diarrhoea rating was assessed by professional individuals who had no prior knowledge of the treatment assignment.

### ETEC shedding

The presence of ETEC in faeces was assessed by detection of virulence genes *k88*, *staP*, and *stb*, using real-time qPCR^35^, as outlined below.

#### Generation of target genes

Target genes from ETEC strain L3.2 were extracted using the GeneJET Genomic DNA Purification Kit (Thermo Scientific) and amplified by PCR using the GoTaq Green Master mix (Promega) according to the manufacturer’s instructions. The primer sequences used for PCR in this study are shown in Table 3. The following conditions were used for amplification: initial denaturation at 94 °C for 5 min, followed by 30 cycles of denaturation for 40 s at 94 °C, annealing at 55 °C for 1 min, and extension for 1.5 min at 72 °C. At each cycle, the extension time was raised 3 s, and the final extension was 5 min at 72 °C. PCR clean-up kits (NucleoSpin) were used to purify the PCR products.

**Table 3:** DNA sequence of PCR primers for virulence genes.

Virulence gene	Primer sequence	Melting temperature (°C)	Product size (bp)	References
*k88*	GTTGGTACAGGTCTTAATGG GAATCTGTCCGAGAATATCA	57	499	Casey and Bosworth ^34^
*staP*	CAACTGAATCACTTGACTCTT TTAATAACATCCAGCACAGG	57	158	
*stb*	TGCCTATGCATCTACACAAT CTCCAGCAGTACCATCTCTA	58	113	

#### DNA ligation and transformation

The purified target genes were ligated individually into the pGEM-T Easy vector (Promega) by T4 DNA ligase (Thermo Scientific) at 22 °C for 30 min. These ligations were used to transform competent *E. coli* DH5a (Yeastern Biotech) using the heat-shock method. Briefly, 50 µl of competent *E. coli* were added to 5 μL of the ligations, incubated on ice for 30 min, and then placed at 42 °C in a water bath for exactly 30 s before being moved into ice for 2 min. Transformed *E. coli* were recovered by adding 1 mL of super optimal broth with catabolite repression (SOC) medium (Thermo Scientific), then incubating at 37 °C for 1.5 h in a shaker incubator at 250 rpm. Using blue-white colony screening, the transformed *E. coli* containing each target gene were plated on LB agar mixed with ampicillin (100 μg/mL), isopropyl-β-d-thiogalactopyranoside (IPGT; 200 mM), and 5-bromo-4-chloro-3-indolyl-β-d-galactopyranoside (X-gal; 50 mg/mL). These plates were incubated for 18 h at 37 °C. Selected white colonies were subjected to PCR to check recombination. A white colony carrying the target gene was transferred to LB broth and cultured for 18 h at 37 °C.

#### Plasmid purification and standard curve construction

For each target gene, the plasmids containing the gene were purified using Plasmid EasyPure (NucleoSpin). The plasmid’s concentration was assessed using Qubit 4 fluorometers with the dsDNA broad-range assay kit (Invitrogen, Thermo Fisher Scientific) and adjusted to 10^9^ copies/μL. The plasmids were subjected to a ten-fold dilution from 10^9^ to 10^2^ copies/μL. A standard curve was constructed using all of the dilutions. The number of copies of each target gene was calculated using the standard curves.

#### Faecal DNA isolation and real-time qPCR

Ten (5 female and 5 male) pigs in each experimental group were randomly selected for faecal collection. DNA was extracted from faeces samples from each experimental group using the Quick-DNA Fecal/Soil Microbe Miniprep Kits (Zymo Research). The qPCR was conducted separately for each virulence gene, with detection using the StepOnePlus Real-Time PCR System with StepOne Software V2.3 (Applied Biosystems) and SYBR Green-Based fluorescent dye. Each faecal DNA sample was tested in triplicate, and the results were averaged and displayed as copy number/μL. The assays utilized 50–200 ng of stool DNA, 8 μL of water, 0.5 μL each of 10 μM forward and reverse primers (Table 3), and 10 μL of PowerUp SYBR Green Master Mix (Applied Biosystems). The real-time qPCR was carried out with the following conditions: initial denaturation at 50 °C for 2 min, 95 °C for 2 min, 40 cycles of denaturation at 95 °C for 15 s, annealing at 55 °C for 15 s, and extension at 72 °C for 1 min. Gene copies were determined by absolute quantification using the standard curve fitted for each 96-well plate. R^2^ values for a linear model fit with cycle threshold (Ct) vs. log dilution of DNA in the standard curve ranged from 1.00 to 0.988.

### Faecal LAB *and E. coli* counts

Faecal samples were obtained from rectal swabbing, which were preserved on ice and transported to the laboratory immediately. Ten (5 females and 5 males) of the 12 pigs in each experimental group were randomly selected for faecal collection. Faecal samples were pooled (1 g) and mixed with normal saline (1:9 w/v). For the determination of LAB and *E.coli* viable cell counts, the supernatants were subjected to serial dilution and plated using the spread plate method at the appropriate dilution onto 3 M Petrifilm Lactic acid bacteria count plates (for LAB) and Rapid *E.coli*/Coliform count plates (3 M) (for *E. coli*). The petrifilms were incubated at 37 °C for 48 hr^36^. Colony counting was done in triplicate, and the CFU/g faeces was calculated.

### Cytokine evaluation

Blood samples were allowed to coagulate in their vacutainer tubes for approximately 10–15 min before being centrifugated at 1,000 × g for 10 min at 4 °C. The serum was aliquoted, transported to the laboratory and stored at − 20 °C^37^. The concentrations of porcine cytokines interleukin 1 alpha (IL-1α), interleukin 6 (IL-6), interleukin 8 (IL-8), tumor necrotic factor alpha (TNFα), and interleukin 10 (IL-10) were individually measured according to the manufacturer’s instructions using the MILLIPLEX MAP Porcine cytokine/chemokine magnetic bead panel (Millipore) in the MAGPIX system with xPONENT software V4.2 (Luminex corporation)^22^. The processes were repeated twice, and the cytokine concentrations were determined as ng/mL unit.

### Pathological analysis

At necropsy, samples of the walls of the duodenum, jejunum, and ileum were collected from each pig and fixed in 10% neutral buffered formalin. Subsequently, the fixed intestine segments were dehydrated in alcohol, cleared in xylene, and embedded in paraffin wax. Sections of 4–6 μm were cut from the blocks and stained with haematoxylin and eosin^38^. The sections were examined for pathological lesions and intestinal morphology. Villus height (VH) and crypt depth (CD) were measured using Motic Images Plus Version 2.0 (Motic), with an average of ten measurements used per sample, and these then were converted into the VH:CD ratio.

### Statistical analysis

All of the experiments were analysed with Prism 9 for macOS version 9.3.1 (350). Effects were considered significant at *P* < 0.05. The mean ± standard error of the mean (SEM) and median with range were used to present the findings. Data analyses between groups were carried out using one-way ANOVA, with comparison of means tested by Tukey’s multiple range tests. The analyses on the combined effect of two variables, including experimental groups and period, were conducted with two-way ANOVA and Tukey’s multiple range tests. Non-normal distributed data were examined by the Kruskal–Wallis test with Dunn’s test for nonparametric pairwise multiple comparisons.

## Supplementary Information

Supplementary Information.
