# Supplementary material for: Microencapsulated probiotic Lactiplantibacillus plantarum and/or Pediococcus acidilactici strains ameliorate diarrhoea in piglets challenged with enterotoxigenic Escherichia coli
Source: Sci Rep. 2022 May 3;12:7210. doi: 10.1038/s41598-022-11340-3 (PMC9065055; doi:10.1038/s41598-022-11340-3)
Supplement: Supplementary file 1 — Supplementary Information. [file 41598_2022_11340_MOESM1_ESM.docx]

Supplementary data

Microencapsulated probiotic *Lactiplantibacillus plantarum* and/or *Pediococcus acidilactici* strains ameliorate diarrhoea in piglets challenged with enterotoxigenic *Escherichia coli*

Pawiya Pupa^a^, Prasert Apiwatsiri^a^, Wandee Sirichokchatchawan^b^, Nopadon Pirarat^c^, Teerawut Nedumpun^a^, David J. Hampson^d^, Nongnuj Muangsin^e^, Nuvee Prapasarakul^a,f*^

Author details:

^a^Department of Microbiology, Faculty of Veterinary Science, Chulalongkorn University, Bangkok 10330, Thailand

^b^College of Public Health Sciences, Chulalongkorn University (CPHS), Bangkok, Thailand

^c^Department of Pathology, Faculty of Veterinary Science, Chulalongkorn University, Bangkok 10330, Thailand

^d^School of Veterinary Medicine, Murdoch University, Perth 6150, Australia

^e^Department of Chemistry, Faculty of Science, Chulalongkorn University, Bangkok 10330, Thailand

^f^Diagnosis and Monitoring Animal Pathogens Research Unit, Chulalongkorn University, Bangkok 10330, Thailand

***Correspondence:**

Nuvee.p@chula.ac.th

| **Ingredient Composition** | **% of dry matter** | |
| --- | --- | --- |
| Corn (7.7 % CP) | 26.75 | |
| Dehull-soybean meal (48.9 % CP) | 18.47 | |
| Broken rice (7.7 % CP) | 22.97 | |
| Full fat soybean (36.0 % CP) | 10.00 | |
| Rice bran, full fat (13.6 % CP) | 5.00 | |
| Fish meal (60 % CP) | 6.00 | |
| Whey powder sweet | 5.00 | |
| Soybean oil | 2.67 | |
| Mono-Dicalcium Phosphate (MDCP; P 18.0 %, Ca 21.8 %) | 1.29 | |
| Limestone (Ca 36.4%) | 0.20 | |
| SP Premix | 0.50 | |
| Pellet binder | 0.30 | |
| Salt | 0.23 | |
| L-Lysine HCl | 0.27 | |
| DL-Methionine | 0.15 | |
| L-Threonine | 0.15 | |
| L-Tryptophan | 0.05 | |
| Total | 100.00 | |
| **Nutrient concentration** |  | **Unit** |
| Standardized ileal digestible Lysine:Metabolisable Energy (SID Lysine:ME) | 3.83 | g/Mcal |
| Crude fat | 7.50 | % |
| Digestible Lysine | 1.33 | % |
| Digestible Methionine | 0.49 | % |
| Digestible Methionine + Cysteine | 0.76 | % |
| Digestible Threonine | 0.83 | % |
| Digestible Tryptophan | 0.27 | % |
| Calcium | 0.82 | % |
| Phosphorus | 0.54 | % |

**Supplementary Table S1.** Ingredient composition and nutrient concentration of the experimental basal diet.


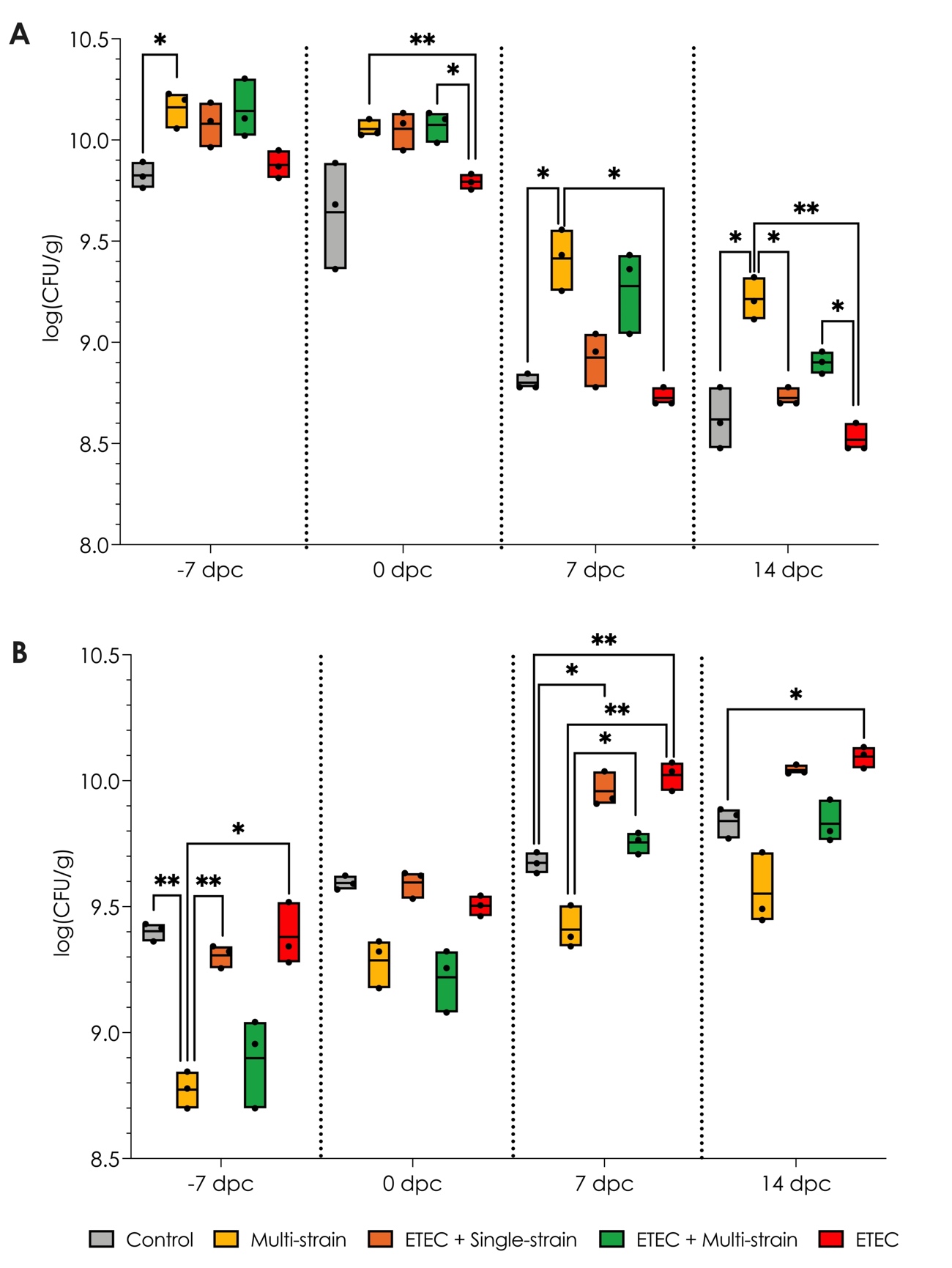


**Supplementary Figure S1.** Faecal bacterial counts in pigs in each experimental group throughout the experiment. A = LAB counts; and B = *E. coli* counts. Data are presented as individual values (black dots) of each replication from bacterial enumerations in each group within the floating bars (min to max), in which the mean values are indicated by the central black horizontal lines. The asterisks represent statistically significant differences (* = *P* < 0.05 and ** = *P* < 0.01). dpc = day post-challenge.


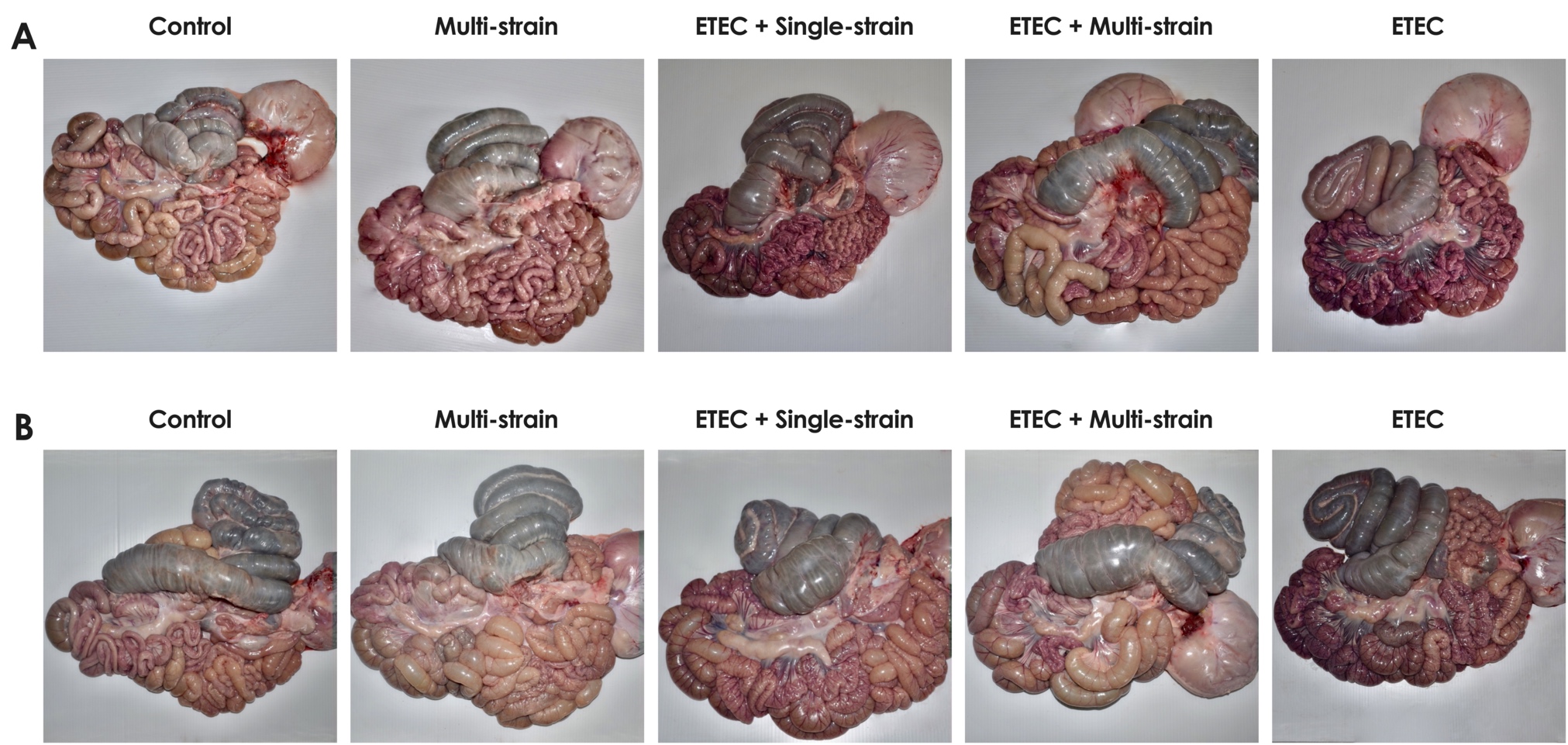


**Supplementary Figure S2.** Gross pathology of the small intestine in piglets after ETEC challenging (5×10^9^ CFU). A = 7 days post challenge; and B = 14 days post challenge.


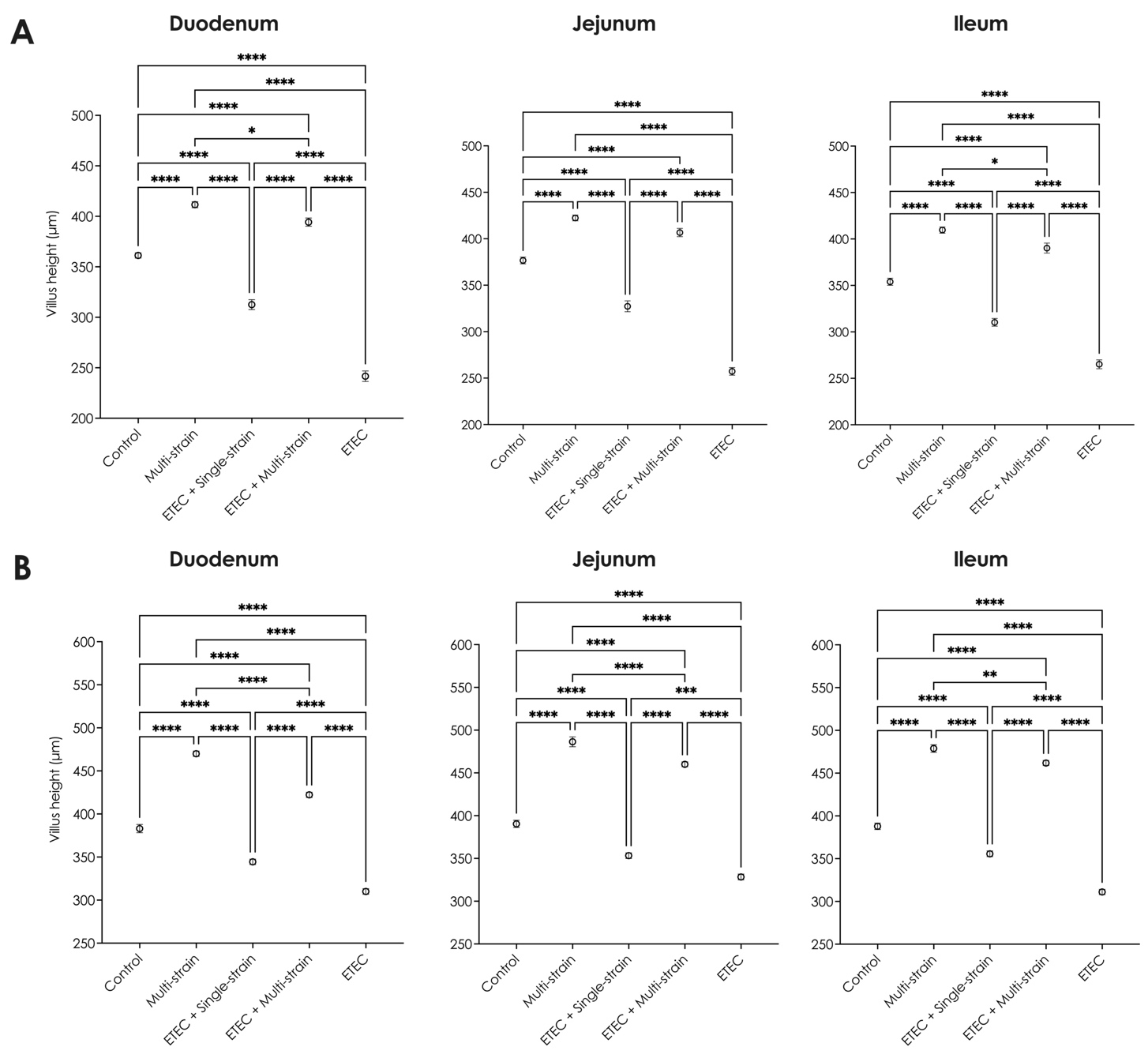


**Supplementary Figure S3.** The villus height (VH) of the small intestine in piglets after ETEC challenge (5×10^9^ CFU). A = 7 days post challenge; and B = 14 days post challenge. Values are presented as mean ± SEM of all replications in each group. The asterisks represent statistically significant differences (* = *P* < 0.05, ** = *P* < 0.01, *** = *P* < 0.001 and **** = *P* < 0.0001).


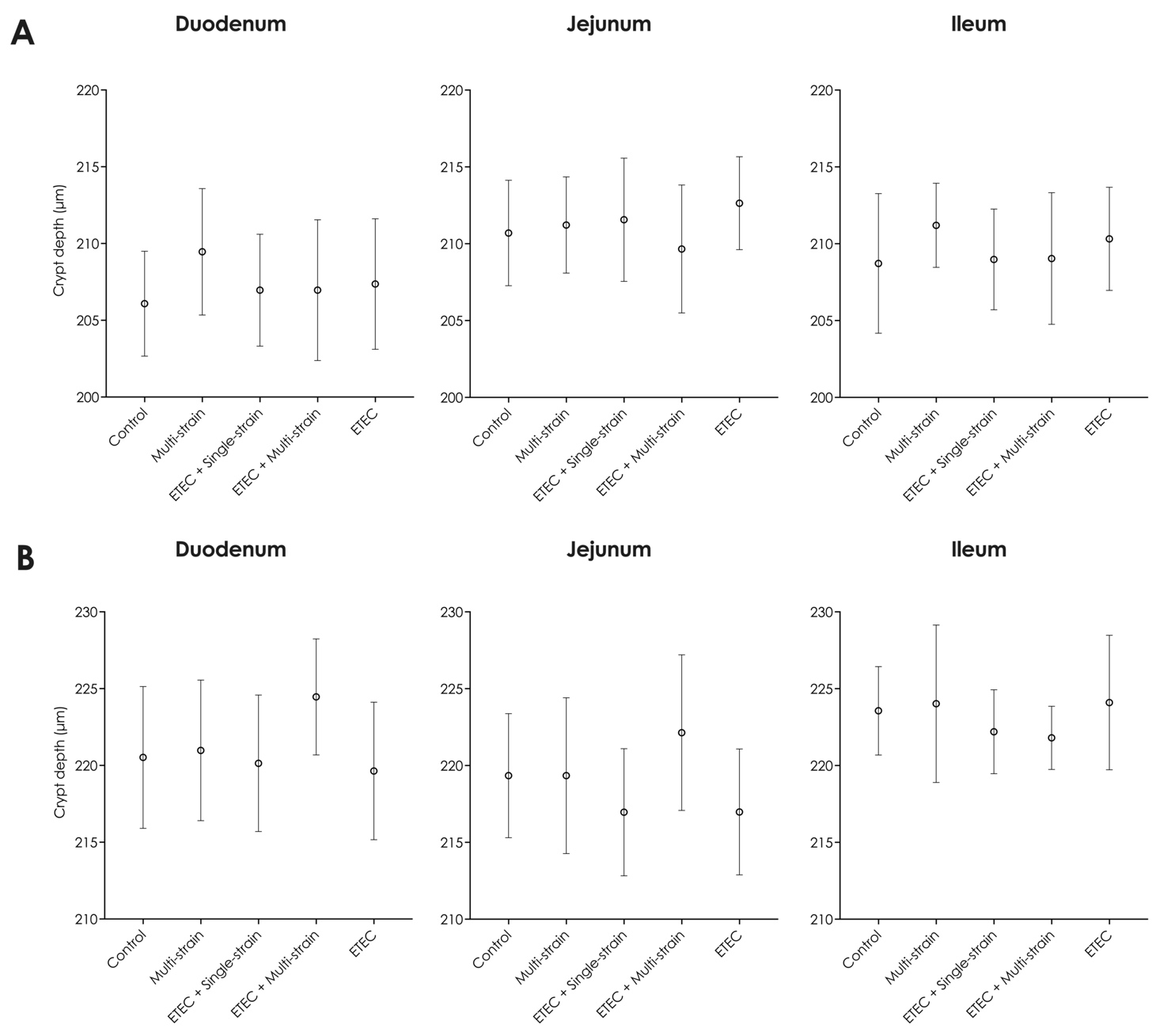


**Supplementary Figure S4.** The crypt depth (CD) of the small intestine in piglets after ETEC challenge (5×10^9^ CFU). A = 7 days post challenge; and B = 14 days post challenge. Values are presented as mean ± SEM of all replications in each group.


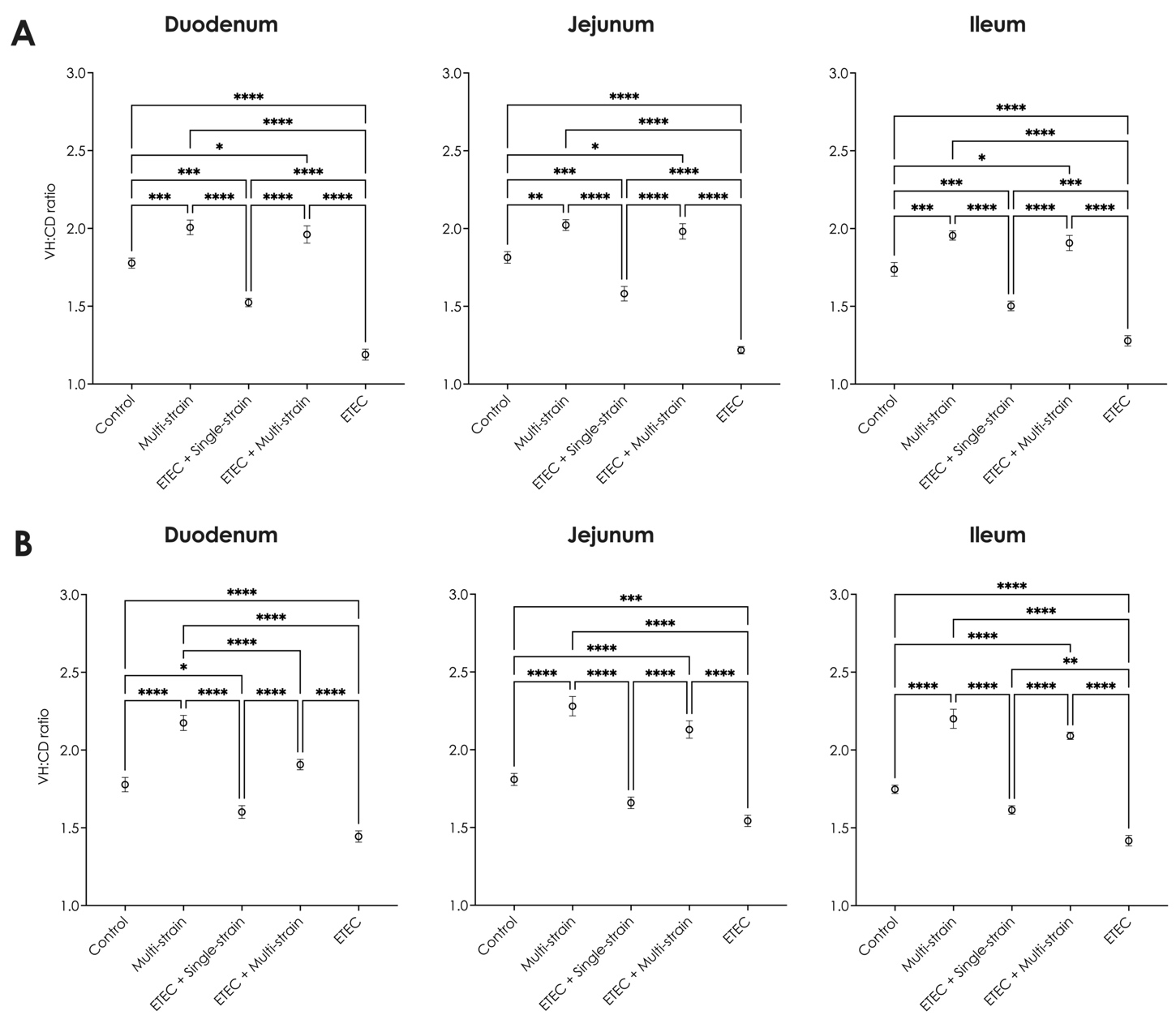


**Supplementary Figure S5.** The VH:CD ratio of the small intestine in piglets after ETEC challenge (5×10^9^ CFU). A = 7 days post challenge; and B = 14 days post challenge. Values are presented as mean ± SEM of all replications in each group. The asterisks represent statistically significant differences (* = *P* < 0.05, ** = *P* < 0.01, *** = *P* < 0.001 and **** = *P* < 0.0001).


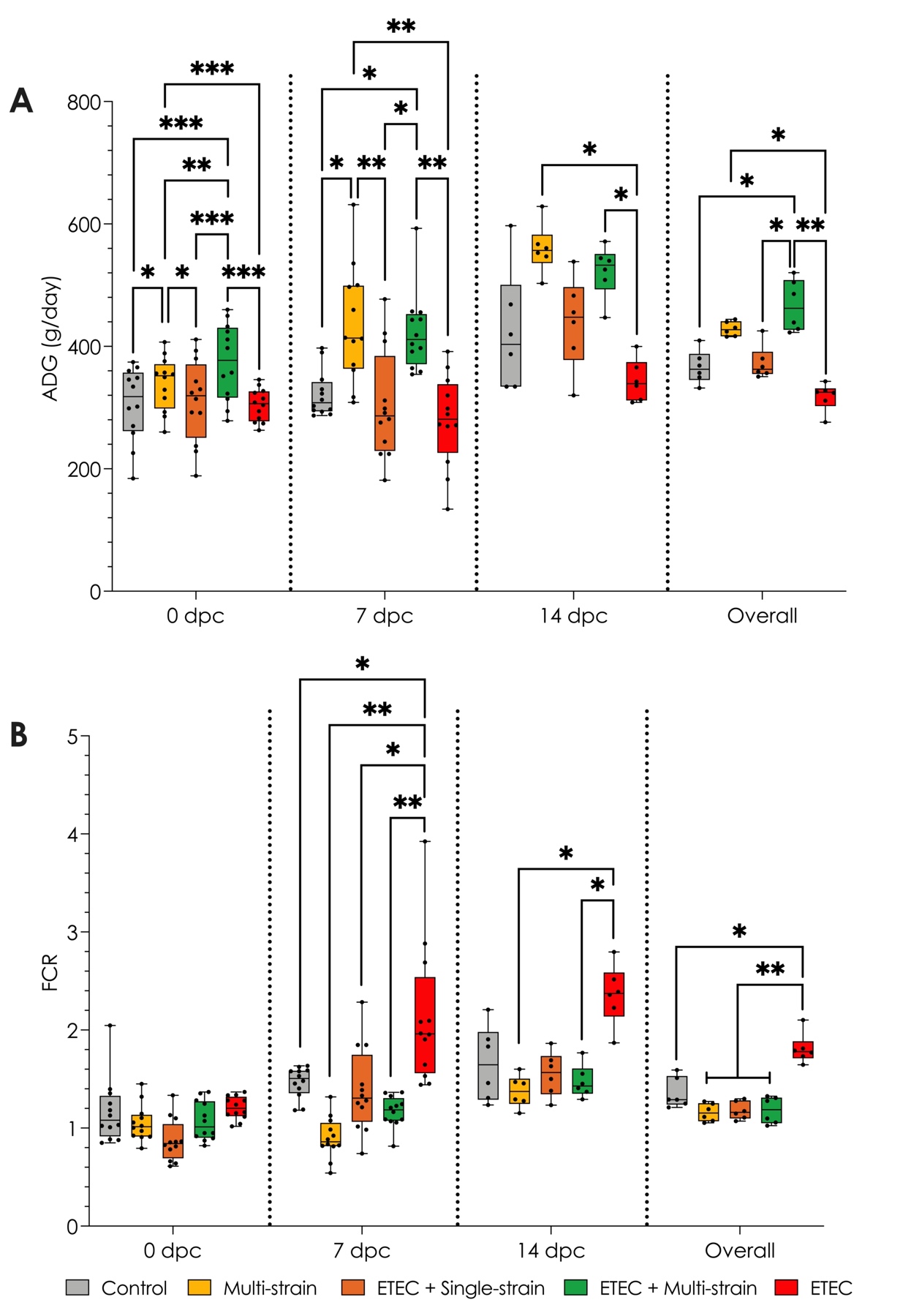


**Supplementary Figure S6.** Growth performance of piglets after ETEC challenge (5×10^9^ CFU). A = Average daily gain (ADG); and B = Feed conversion ratio (FCR). Data are presented as individual values (black dots) of each animal in each group with the box-and-whisker plot in which the median values are indicated by the central black horizontal lines; the boxes extend from 25th to 75th percentiles, and the whiskers extend down to the minimum and up to the maximum value. The asterisks represent statistically significant differences (* = *P* < 0.05, ** = *P* < 0.01 and *** = *P* < 0.001). dpc = day post-challenge.


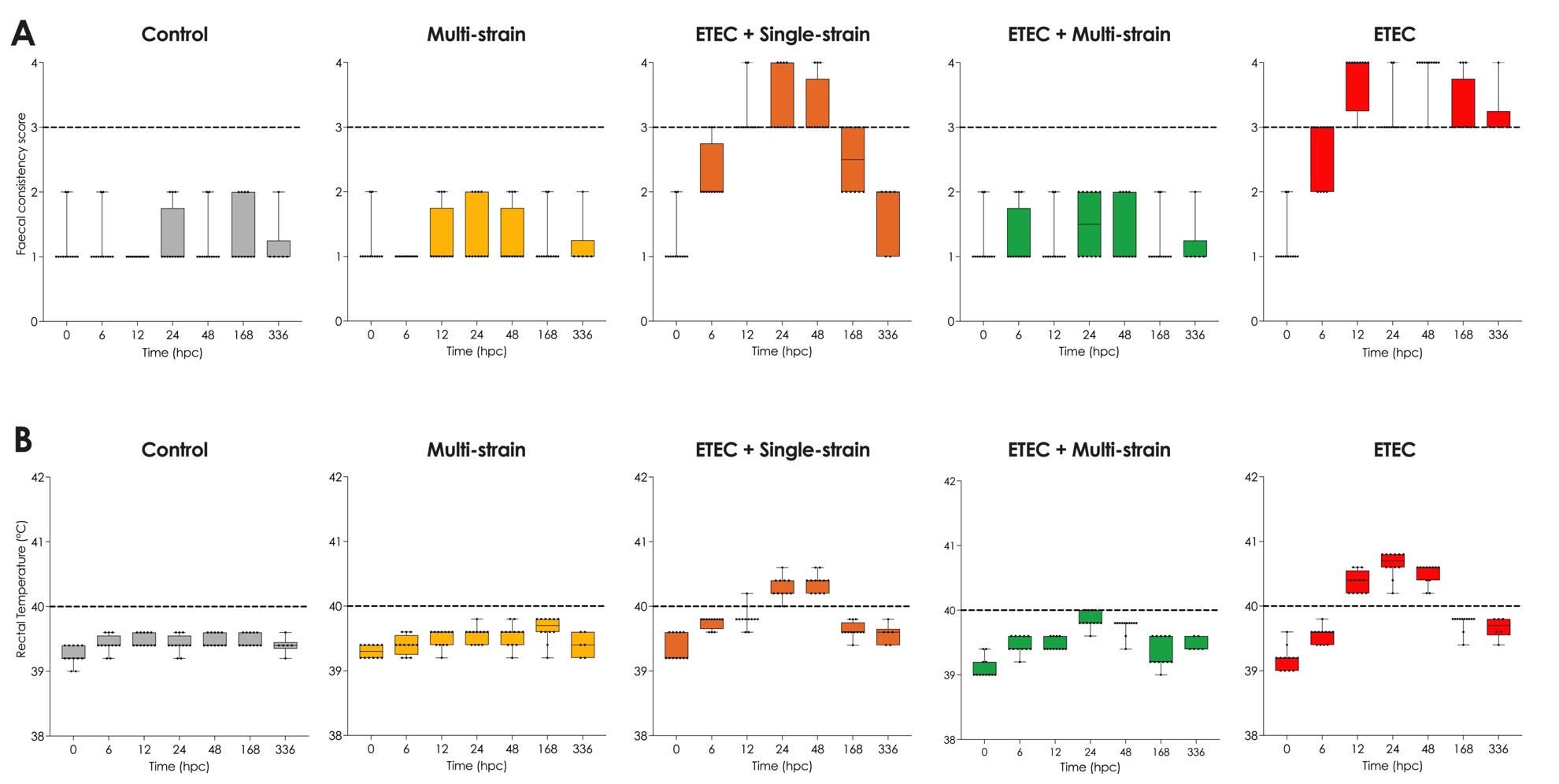


**Supplementary Figure S7.** Clinical signs in piglets after ETEC challenge (5×10^9^ CFU). A = Faecal consistency score (FCS); and B = Rectal temperature (RT). Data are presented as individual values (black dots) of each animal in each group with the box-and-whisker plot in which the median values are indicated by the central black horizontal lines; the boxes extend from 25th to 75th percentiles, and the whiskers extend down to the minimum and up to the maximum value. FCS and RT, which on or over the dashed line, indicated diarrhoea signs and fever in the piglets. hpc = hour post-challenge.


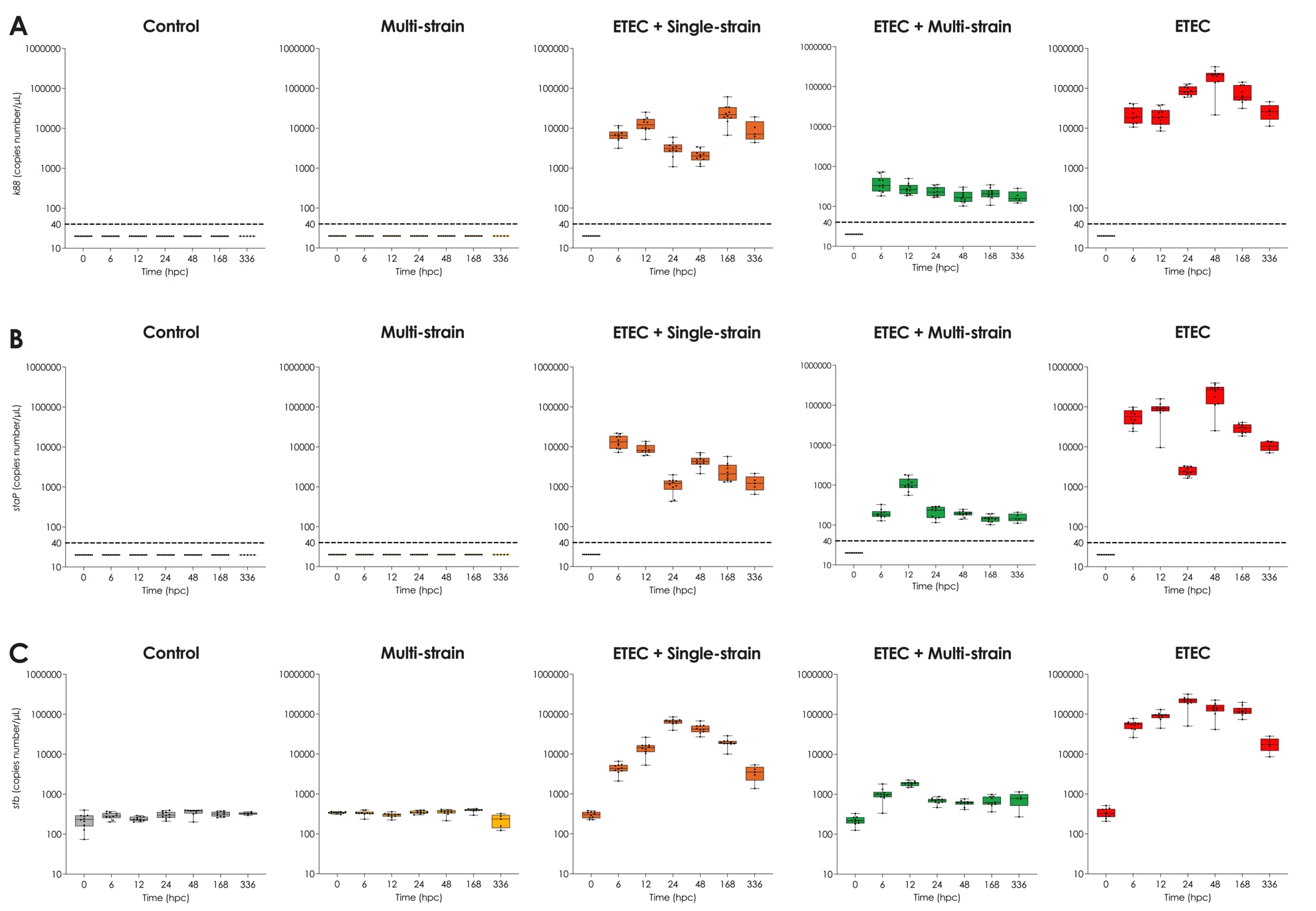


**Supplementary Figure S8.** Virulence gene detection in piglets’ faeces after ETEC challenge (5×10^9^ CFU). A = *k88*; B = *staP*; and C = *stb*. Data are presented as individual values (black dots) of each animal in each group with the box-and-whisker plot in which the median values are indicated by the central black horizontal lines; the boxes extend from 25th to 75th percentiles, and the whiskers extend down to the minimum and up to the maximum value. Values under the dashed line indicate the under-determination of gene copies number (≤ 40 copies number/μL). hpc = hour post-challenge.


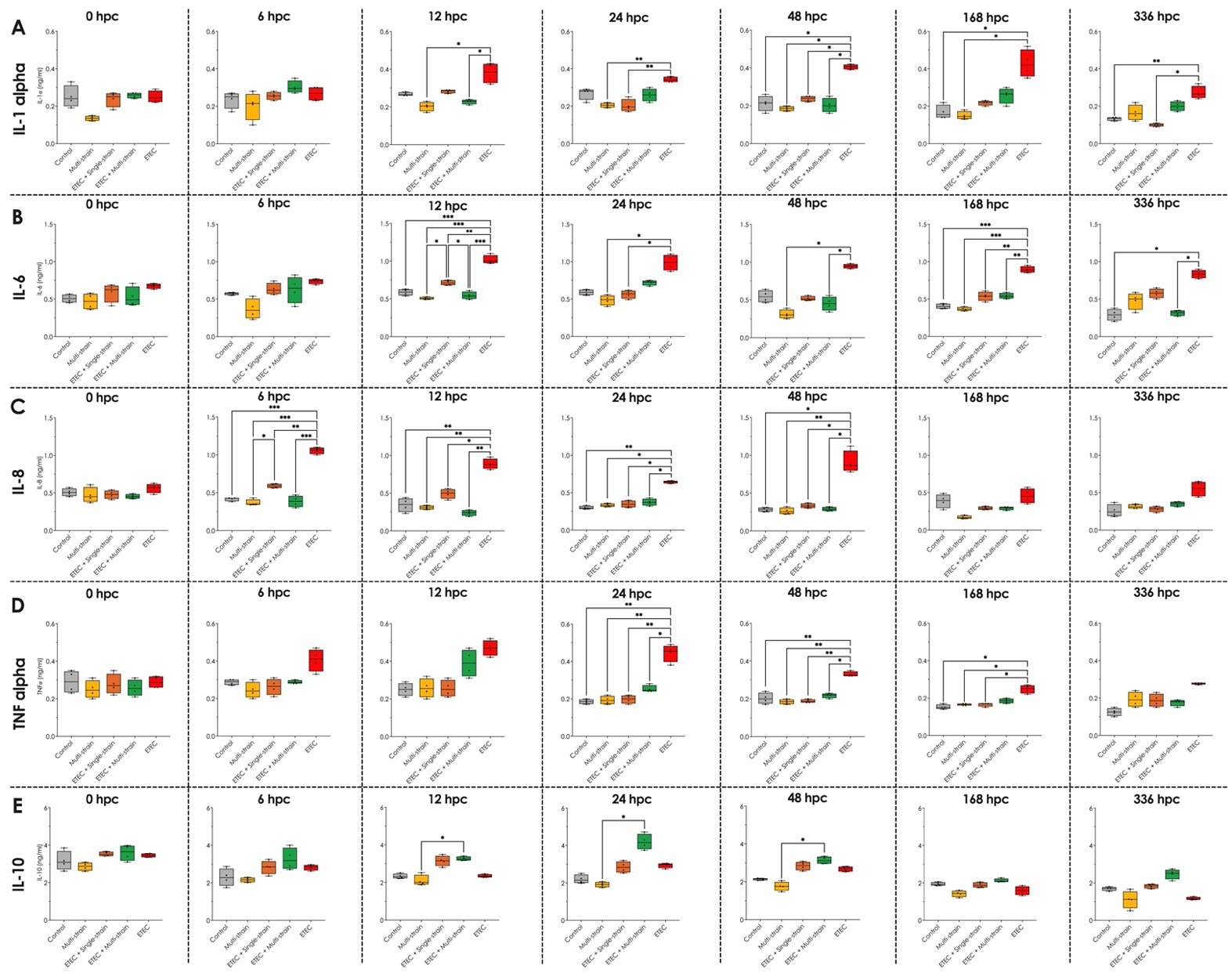


**Supplementary Figure S9.** Serum cytokine concentrations at different time point in piglets after ETEC challenge (5×10^9^ CFU). A = IL-1α; B = IL-6; C = IL-8; D = TNFα; and E = IL-10. Data are presented as individual values (black dots) for each animal in each group with the box-and-whisker plot in which the median values are indicated by the central black horizontal lines; the boxes extend from 25th to 75th percentiles, and the whiskers extend down to the minimum and up to the maximum value. The asterisks represent statistically significant differences (* = *P* < 0.05, ** = *P* < 0.01 and *** = *P* < 0.001). hpc = hour post-challenge.


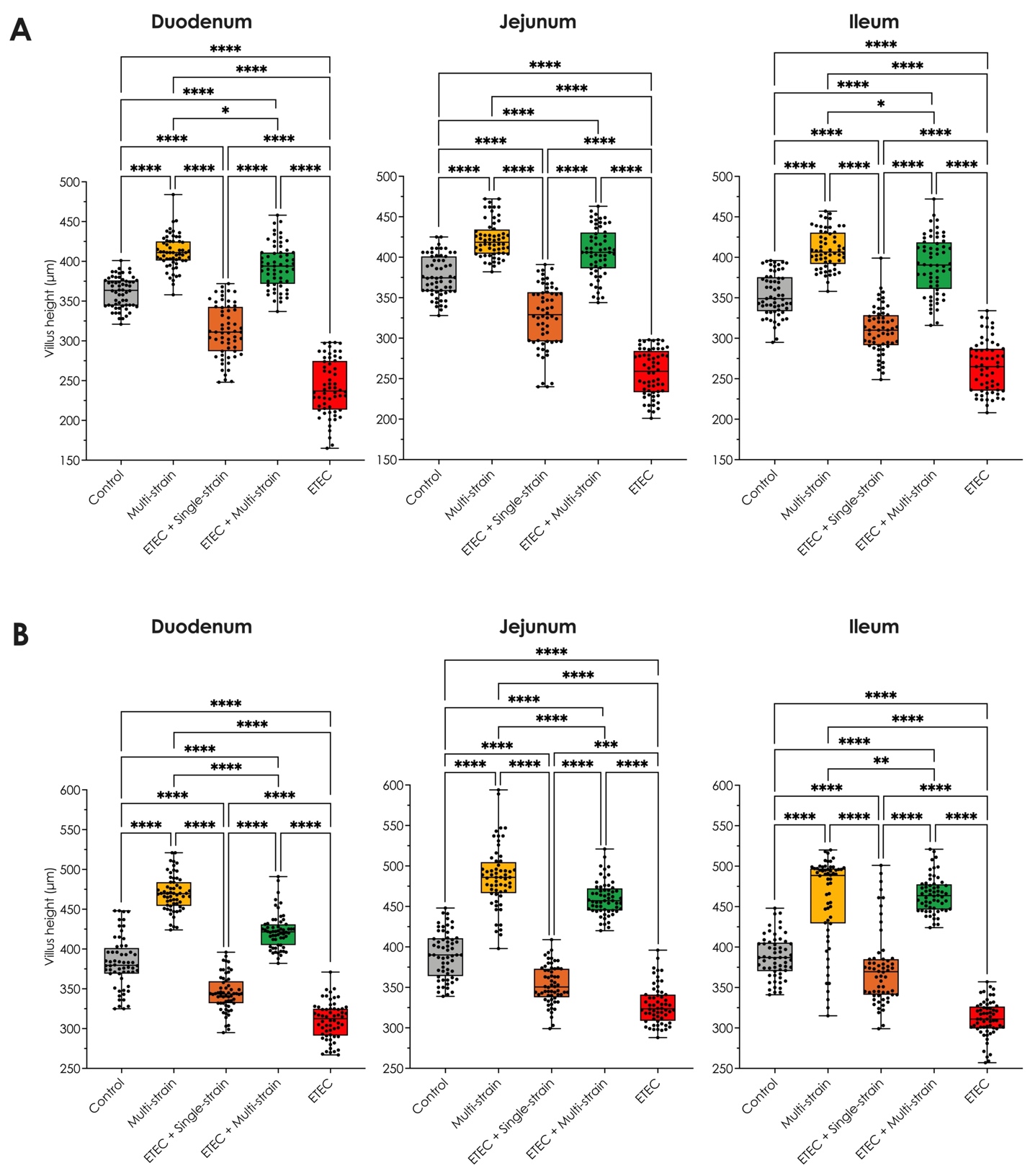


**Supplementary Figure S10.** The villus height (VH) of the small intestine in piglets after ETEC challenge (5×10^9^ CFU). A = 7 days post challenge; and B = 14 days post challenge. Data are presented as individual values (black dots) of each measurement from each animal in each group with the box-and-whisker plot in which the median values are indicated by the central black horizontal lines; the boxes extend from 25th to 75th percentiles, and the whiskers extend down to the minimum and up to the maximum value. The asterisks represent statistically significant differences (* = *P* < 0.05, ** = *P* < 0.01, *** = *P* < 0.001 and **** = *P* < 0.0001).


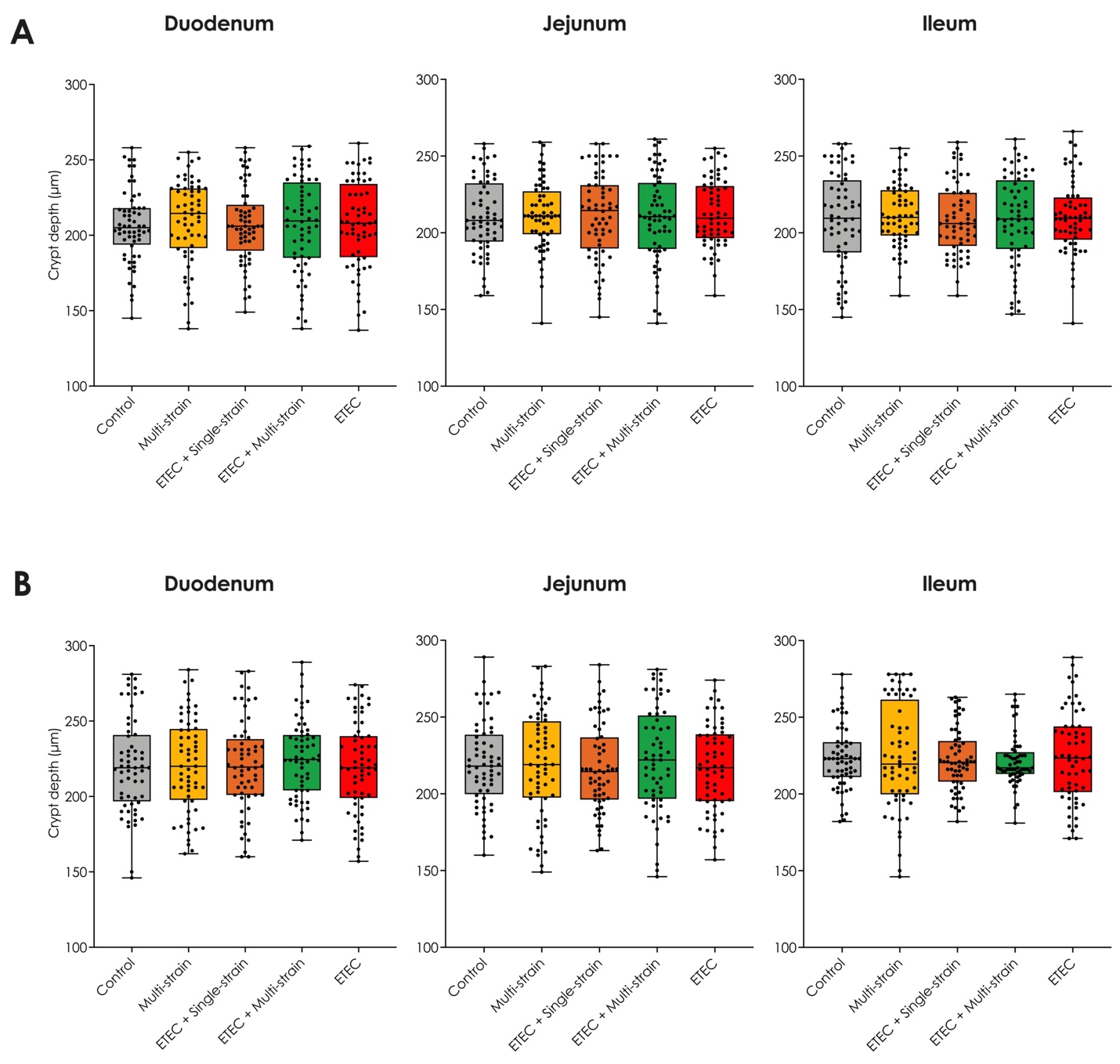


**Supplementary Figure S11.** The crypt depth (CD) of the small intestine in piglets after ETEC challenge (5×10^9^ CFU). A = 7 days post challenge; and B = 14 days post challenge. Data are presented as individual values (black dots) of each measurement from each animal in each group with the box-and-whisker plot in which the median values are indicated by the central black horizontal lines; the boxes extend from 25th to 75th percentiles, and the whiskers extend down to the minimum and up to the maximum value.


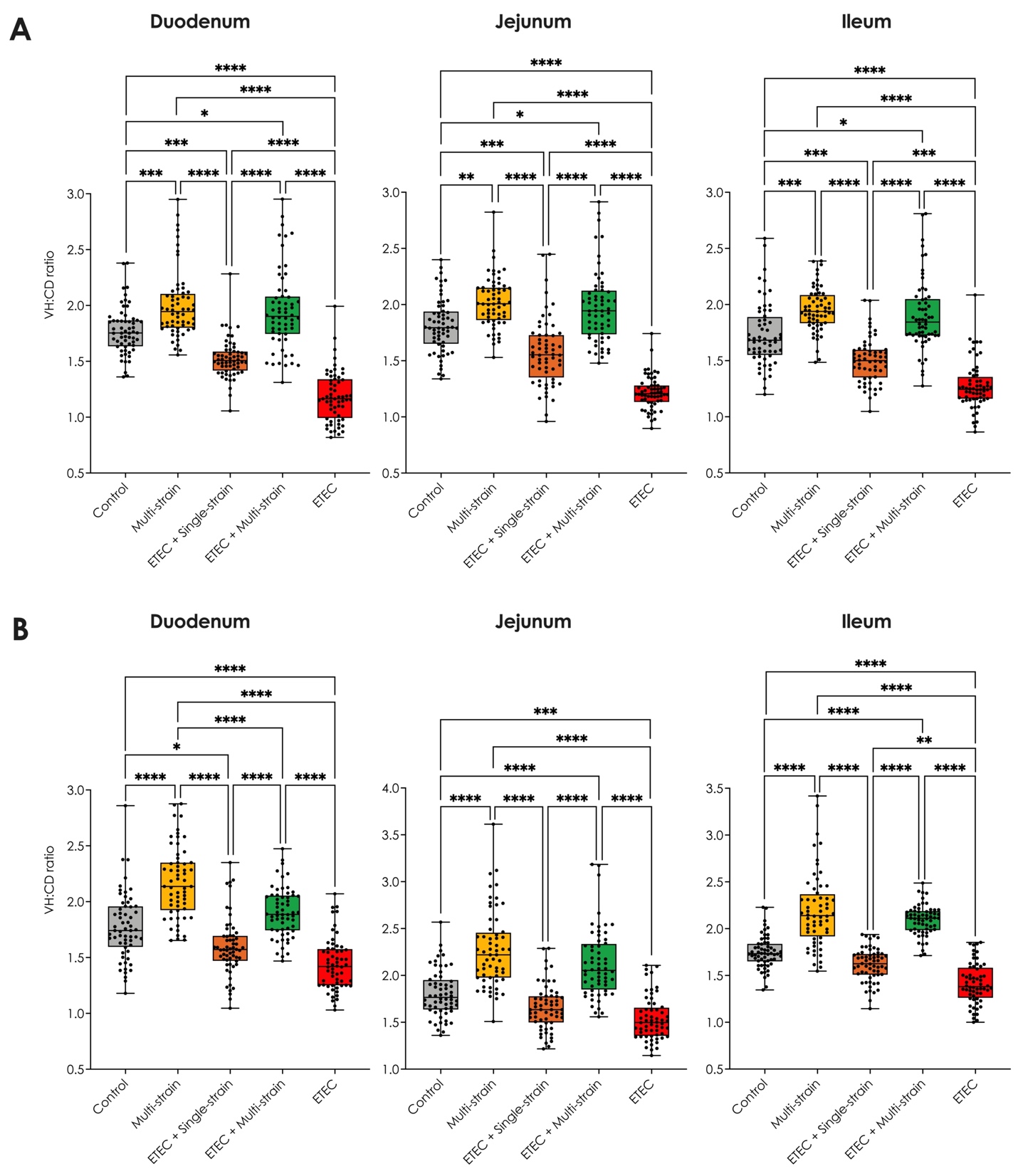


**Supplementary Figure S12.** The VH:CD ratio of the small intestine in piglets after ETEC challenge (5×10^9^ CFU). A = 7 days post challenge; and B = 14 days post challenge. Data are presented as individual values (black dots) of each measurement from each animal in each group with the box-and-whisker plot in which the median values are indicated by the central black horizontal lines; the boxes extend from 25th to 75th percentiles, and the whiskers extend down to the minimum and up to the maximum value. The asterisks represent statistically significant differences (* = *P* < 0.05, ** = *P* < 0.01, *** = *P* < 0.001 and **** = *P* < 0.0001).
